# COVID-19 Vaccination Among Diverse Population Groups in the Northern Governorates of Iraq

**DOI:** 10.3389/ijph.2023.1605736

**Published:** 2023-11-28

**Authors:** Mohammed Ibrahim Mohialdeen Gubari, Falah Wadi, Khalid Anwar Hama-Ghareeb, Fatah H. Fatah, Mostafa Hosseini, Karzan Rafiq Wale, David Hipgrave, Sazan Raouf Ali, Shaho Osman Mahmood, Waleed Ezzat Khadium, Hayman Hasan Mohammed, Sara Feal Jaafer, Najeeb Mohammed Al Saadi, Kardar Anwar Mohammed, Shireen Salih Saeed, Mohammad Yousif Mohammad, Waleed Hamid Khudhur, Mohammed Wael Saleh, Yousra Saadi Sheat, Khaldoon Khaleel Ibrahim, Marwa Nabeil Salah, Abdulmonem Hazim Abdullah, Dashne Shamall Omer, Rafeeq Naseraldeen Ghafur, Kashma Ali Mustafa, Aryan Othman Faraj, Trifa Bhjat Ali, Kamal Aziz Enayat, Ronak Assi Wahab, Ibrahim Ahmed Ali Al-Jaf, Nazanin Hama Hama Amin, Dina Dlshad Jaff, Aram Mohammed Bra, Shwan Kanabi Ahmed, Banaz Nabi Rasool, Fatimah Khalis Jamal, Tango Dhahir Mohammed, Maryam Arab Azeez

**Affiliations:** ^1^ Department of Family and Community Medicine, University of Sulaimani, Sulaimani, Iraq; ^2^ United Nations International Children’s Emergency Fund (UNICEF), Baghdad, Iraq; ^3^ Research Department, General Directorate of Health, Sulaimani, Iraq; ^4^ Department of Epidemiology and Biostatistics, School of Public Health, Tehran University of Medical Science (TUMS), Tehran, Iran; ^5^ Directorate of Preventive Health Department, General Directorate of Health, Sulaimani, Iraq; ^6^ United Nations International Children’s Emergency Fund (UNICEF), Iraq Country Office, Baghdad, Iraq; ^7^ Department of Planning, Directorate of Health, Sulaimani, Iraq; ^8^ Surgical Teaching Hospital, Sulaimani, Iraq; ^9^ Public Health Department, Kirkuk Health Directorate, Kirkuk, Iraq; ^10^ Expanded Program on Immunization Section—Mother and Child Healthcare Department, Directorate of Preventive Health Affairs, Duhok, Iraq; ^11^ Public Health Department, Ninawa Directorate of Health, Mosul, Iraq; ^12^ Ministry of Health, Sulaimani, Iraq; ^13^ Erbil General Directorate of Health, Erbil, Iraq

**Keywords:** coronavirus disease 2019 (COVID-19), COVID-19 vaccine, vaccine hesitancy, sociodemographic factors, vaccination acceptance

## Abstract

**Objectives:** The present study was carried out to investigate COVID-19 vaccination coverage among populations of internally displaced persons (IDPs), refugees, and host communities in northern Iraq and the related underlying factors.

**Methods:** Through a cross-sectional study conducted in five governorates in April–May 2022, 4,564 individuals were surveyed. Data were collected through an adapted questionnaire designed to gather data on participants.

**Results:** 4,564 subjects were included (59.55% were 19–45 years old; 54.51% male). 50.48% of the participants (51.49% of host communities, 48.83% of IDPs, and 45.87% of refugees) had been vaccinated with at least one dose of COVID-19 vaccine. 40.84% of participants (42.28% of host communities, 35.75% of IDPs, and 36.14% of refugees) had been vaccinated by two doses, and 1.56% (1.65% of host communities, 0.93% of IDPs, and 1.46% of refugees) were vaccinated with three doses.

**Conclusion:** Sociodemographic factors including age, gender, education, occupation, and nationality could affect vaccination coverage. Moreover, higher acceptance rate of vaccination is associated with belief in vaccine safety and effectiveness and trust in the ability of the vaccine to prevent complications.

## Introduction

As a worldwide pandemic, coronavirus disease 2019 (COVID-19) is referred to as a public health emergency of international concern [1]. Iraq’s first confirmed cases of severe acute respiratory syndrome coronavirus 2 (SARS-CoV-2) infections were reported in Najaf governorate in February 2020. By April, there was a sharp rise in the number of confirmed cases in Baghdad, Basra, Erbil, Sulaymaniyah, and Karbala, resulting in a great burden on the social and mental health all over Iraq [2, 3].

So far, over 2,460,844 cases and 25,356 deaths due to COVID-19 have been reported in Iraq till 20 October 2022 [4]. Control measures such as containment measures, mask mandate and social distancing, case detection, and tracing were taken to reduce the spread of the infection [5]. Despite the potential effectiveness of such strategies, successful control of severe COVID-19 only became possible with the development of safe and effective vaccines. Zhen et al., in a meta-analysis of 51 studies, indicated that Pfizer-BioNTech and Moderna vaccines had observed effectiveness of 91.2% and 98.1% against infection [6].

As of 11 October 2022, Iraqis have received 19.3 million doses of COVID-19 vaccine, and 18.8% of the population (7.57 million people) are fully vaccinated [4], while some neighboring countries such as Iran and Egypt have fully vaccinated almost 70% of their population [7]. Studies have shown that vaccine rollouts have been only partially successful. In a review, Troiano and Nardi have demonstrated that a maximum of 77.6% of the general population have declared they will accept the COVID-19 vaccine; with the number decreasing according to the socioeconomic status of the population. Factors such as working status, religiosity, political views, and even gender have been proposed to influence vaccine acceptance [8]. Sherman et al. have indicated that over 75% of participants in an online survey completed in the United Kingdom were willing to receive COVID-19 vaccine, with others believing that vaccination was only needed by those at serious risk of illness, or is just a means for manufacturers to make money [9]. Another study conducted in Italy revealed that over 90% of participants desired to be vaccinated [10]. Studies in low/middle-income countries demonstrated widespread conspiracy theories and reluctance to receive COVID-19 vaccines [11, 12].

It is important to understand socio-demographic factors that affect vaccine decision-making [11]. Moreover, successful vaccine rollout depends on identifying and mitigating factors associated with vaccine hesitancy and knowing the sociodemographic factors associated with vaccine hesitancy would aid health decision-makers in adopting specific measures to prompt vaccination among targeted groups [12]. While COVID-19 vaccine acceptance has been documented as low among Kurdish people (13%) [13], so far, no studies have focused on the factors associated with vaccination coverage among Iraqis. This study reports COVID-19 vaccination coverage amongst populations of host communities, internally displaced persons (IDPs) and refugees in five governorates of northern Iraq. In addition, it reports factors associated with vaccination acceptance in the areas surveyed.

## Methods

### Study Design and Setting

We conducted a cross-sectional study over 4 weeks in April–May 2022 in five districts of Sulaimaniyah, Erbil, Dahuk, Kirkuk, and Ninawa.

### Study Sample and Sampling Method

The study sample comprised different sub-populations (the general population, IDPs, and refugees) in each governorate. To select the sample, every household in the selected communities was identified on a sketch map and the household list of each area under study. From these lists a small number of households were randomly selected to participate using a household form 550 households were chosen following the recommendations by WHO [14] and finally 4,564 respondents from all governorates were recruited for the purpose of the present study.

### Data Collection Procedure

The target population in the present study included Iraqi population who were able to read or understand Arabic and/or Kurdish (official in the regions). All people aged 12 years and older and living in the five governorates were considered eligible. COVID-19 vaccination coverage was estimated overall, and in each governorate, stratified by subpopulation. This estimation involved conducting essentially five separate surveys, and then combining the results in a weighted fashion to estimate regional vaccination coverage. Data were collected using Kobo Toolbox. Supplementary Material S1 provides a more detailed explanation of sampling method and data collection procedures.

### Questionnaire

Required data were gathered using a 16-item questionnaire originally developed in Malaysia [15]. The questionnaire was adapted by a team of public health specialists at HAEC and reviewed by a technical team at UNICEF and the ministry of health. It collected data on the respondents’ sociodemographic characteristics, medical history, source of information regarding COVID-19, vaccination coverage, number of doses, and factors influencing COVID-19 vaccine refusal.

### Data Analysis Procedure

The collected data were analyzed using STATA 17.0. For this purpose, descriptive analysis was employed for sociodemographic and categorical data, and analytical statistics were used for the variables associated with COVID-19 vaccine coverage. We assessed the influential factors of COVID-19 vaccination using univariate ordinal logistic regression. Then, all the factors that had a *p*-value less than 0.1 in univariate analyses were entered into a multivariate ordinal logistic regression to identify independent factors affecting vaccine coverage. Additional analyses were performed to assess the influential factors of COVID-19 vaccination in each sub-population (host communities, IDPs, refugees). A *p*-value of below 0.05 and a confidence interval of 95% were considered statistically significant.

### Ethical Considerations

The research was approved by the General Directorate of Health’s ethical committee under reference number HR022,27, and informed consents were obtained from the participants. All participants provided informed consent and no identifying data was used in the data analysis.

## Results

### Participants’ Characteristics

Data of 4,564 subjects were included in this study consisting of 3,519 subjects from host communities, 428 IDPs and 617 refugees. Of all the participants, 59.55% were 19–45 years old and 54.51% were male; 28.59% lived in Ninawa, 27.39% in Erbil, and 24.45% in Sulaymaniyah. Most of participants (77.1%) lived as permanent residents. Kurds (59.79%) were the largest ethnical group, followed by Arabs (33.57%) and Turkman (5.26%). Most (95.46%) were Muslims, and 69.89% were married. Occupation varied widely; 14.77% were students. Table 1 demonstrates the baseline characteristics of all the included subjects.

**TABLE 1:** Distribution of baseline characteristics of all participants according to number of COVID-19 vaccination doses (Iraq April–May 2022).

Variables	COVID-19 vaccination status				Total (%)	OR^a^ (95% CI)
	No vaccination	One dose	Two doses	Three doses		
Age group (year)						
12 to 18	363 (79.26)	32 (6.99)	63 (13.76)	0 (0)	458 (10.04)	Ref.
19 to 45	1,304 (47.98)	244 (8.98)	1,137 (41.83)	33 (1.21)	2,718 (59.55)	0.24 (0.19, 0.3)
46 to 65	438 (40.67)	80 (7.43)	527 (48.93)	32 (2.97)	1,077 (23.60)	0.17 (0.13, 0.22)
65 to 98	155 (49.84)	13 (4.18)	137 (44.05)	6 (1.93)	311 (6.81)	0.23 (0.17, 0.32)
Gender						
Male	1,072 (43.09)	217 (8.72)	1,146 (46.06)	53 (2.13)	2,488 (54.51)	Ref.
Female	1,188 (57.23)	152 (7.32)	718 (34.59)	18 (0.87)	2,076 (45.49)	1.75 (1.56, 1.96)
Governate						
Erbil	600 (48.00)	122 (9.76)	498 (39.84)	30 (2.40)	1,250 (27.39)	Ref.
Sulaimani	721 (64.61)	49 (4.39)	342 (30.65)	4 (0.36)	1,116 (24.45)	1.88 (1.6, 2.21)
Duhok	159 (34.79)	22 (4.81)	269 (58.86)	7 (1.53)	457 (10.01)	0.54 (0.44, 0.67)
Kirkuk	133 (30.50)	38 (8.72)	259 (59.40)	6 (1.38)	436 (9.55)	0.5 (0.41, 0.62)
Ninawa	647 (49.58)	138 (10.57)	496 (38.01)	24 (1.84)	1,305 (28.59)	1.09 (0.94, 1.26)
Nationality						
Kurd	1,384 (50.71)	198 (7.26)	1,106 (40.53)	41 (1.50)	2,729 (59.79)	Ref.
Arab	719 (46.93)	146 (9.53)	643 (41.97)	24 (1.57)	1,532 (33.57)	0.9 (0.8, 1.01)
Assyrian	16 (32.65)	3 (6.12)	28 (57.14)	2 (4.08)	49 (1.07)	0.45 (0.26, 0.79)
Turkman	135 (56.25)	22 (9.17)	80 (33.33)	3 (1.25)	240 (5.26)	1.3 (1, 1.68)
Other	6 (42.86)	0 (0)	7 (50.00)	1 (7.14)	14 (0.31)	0.55 (0.19, 1.63)
Religion						
Muslim	2,170 (49.80)	358 (8.22)	1,763 (40.46)	66 (1.51)	4,357 (95.46)	Ref.
Yazedy	57 (46.72)	7 (5.74)	58 (47.54)	0 (0)	122 (2.67)	0.87 (0.61, 1.22)
Christian	32 (39.02)	4 (4.88)	41 (50.00)	5 (6.10)	82 (1.80)	0.55 (0.36, 0.86)
Other	1 (33.33)	0 (0)	2 (66.67)	0 (0)	3 (0.07)	0.45 (0.05, 4.29)
Marital status						
Married	1,457 (45.67)	254 (7.96)	1,419 (44.48)	60 (1.88)	3,190 (69.89)	Ref.
Single	715 (59.63)	102 (8.51)	375 (31.28)	7 (0.58)	1,199 (26.27)	1.8 (1.58, 2.06)
Divorced	85 (53.46)	7 (4.40)	63 (39.62)	4 (2.52)	159 (3.48)	1.27 (0.93, 1.73)
Other	3 (18.75)	6 (37.50)	7 (43.75)	0 (0)	16 (0.35)	0.7 (0.3, 1.63)
Education						
Illiterate	635 (60.13)	80 (7.58)	336 (31.82)	5 (0.47)	1,056 (23.14)	Ref.
Diploma or less	1,337 (54.15)	196 (7.94)	908 (36.78)	28 (1.13)	2,469 (54.10)	0.78 (0.67, 0.90)
University	288 (27.72)	93 (8.95)	620 (59.67)	38 (3.66)	1,039 (22.77)	0.26 (0.22, 0.31)
Occupation						
Health and medical fields	36 (12.08)	12 (4.03)	231 (77.52)	19 (6.38)	298 (6.53)	Ref.
Office worker	103 (27.91)	30 (8.13)	223 (60.43)	13 (3.52)	369 (8.09)	2.75 (1.96, 3.87)
Non-office worker	239 (42.08)	79 (13.91)	241 (42.43)	9 (1.58)	568 (12.44)	5.78 (4.22, 7.91)
Military and security	35 (14.17)	17 (6.88)	193 (78.14)	2 (0.81)	247 (5.41)	1.55 (1.07, 2.27)
Student	429 (63.74)	70 (10.40)	172 (25.56)	2 (0.30)	673 (14.75)	13.27 (9.69, 18.18)
Retired	65 (38.69)	7 (4.17)	89 (52.98)	7 (4.17)	168 (3.68)	3.87 (2.57, 5.81)
Others	1,353 (60.37)	154 (6.87)	715 (31.91)	19 (0.85)	2,241 (49.10)	10.78 (8.09, 14.36)
Health status						
Positive chronic disease	476 (47.70)	73 (7.31)	420 (42.08)	29 (2.91)	998 (21.87)	Ref.
Healthy	1,784 (50.03)	296 (8.30)	1,444 (40.49)	42 (1.18)	3,566 (78.13)	1.15 (1, 1.32)

^a^ Based on univariate ordinal logistic regression.

OR, odds ratio; CI, confidence interval; Ref., reference category.

### COVID-19 Vaccination Coverage

**FIGURE 1:**
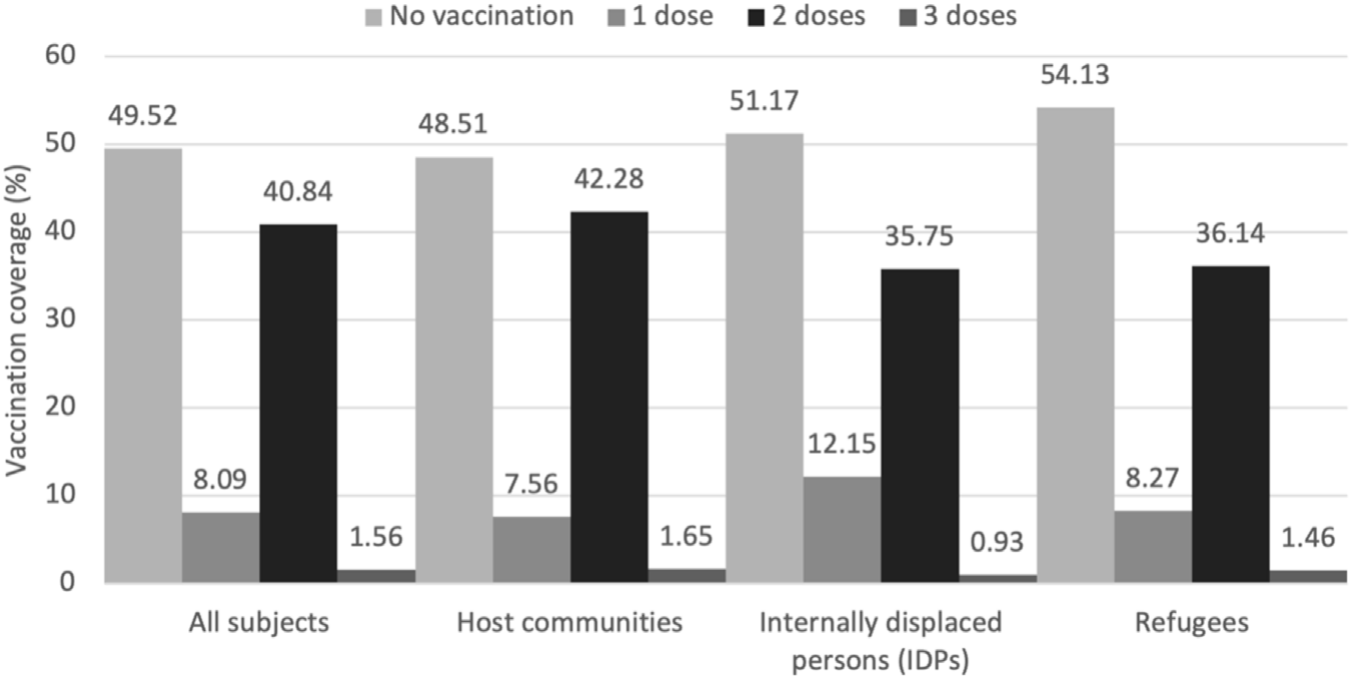
Distribution of COVID-19 vaccination coverage in review (Iraq April–May 2022).

The results revealed that 50.48% of all the participants had been vaccinated by at least one dose of COVID-19 vaccine, while 49.5% had not vaccinated (Figure 1). 40.84% of participants had been vaccinated by two doses, 8.09% by one dose and 1.56% by three doses.

### Influential Factors of COVID-19 Vaccination Coverage

Univariate ordinal logistic regression showed that COVID-19 vaccination coverage was significantly associated with age, gender, place of residence, nationality, religion, marital status, education, occupation and health status (Table 1). The possible attitudinal barriers of COVID-19 vaccination coverage included fear of being unsafe, the vaccine is not effective, COVID-19 is not dangerous, fear of infection following vaccination, inappropriate attitude against the principle of vaccination in general, religious reasons, believing in traditional and local medicine, other reasons (Table 2).

**TABLE 2:** Distribution of possible barriers of COVID-19 vaccination coverage according to number of doses in all participants (Iraq April–May 2022).

Variables	COVID-19 vaccination status				Total (%)	OR^a^ (95% CI)
	No vaccination	One dose	Two doses	Three doses		
Side effects						
No	1,771 (43.46)	369 (9.05)	1,864 (45.74)	71 (1.74)	4,075 (89.27)	Ref.
Yes	489 (100)	0 (0)	0 (0)	0 (0)	489 (10.71)	34.14 (−20,286, 20,285)^b^
Unsafe						
No	1,573 (41.69)	265 (7.02)	1,864 (49.40)	71 (1.88)	3,773 (82.65)	Ref.
Yes	687 (86.85)	104 (13.14)	0 (0)	0 (0)	791 (17.33)	10.75 (8.67, 13.32)
Not effective						
No	1,999 (46.82)	335 (7.79)	1,864 (43.66)	71 (1.66)	4,269 (93.52)	Ref.
Yes	261 (88.47)	34 (11.52)	0 (0)	0 (0)	295 (6.46)	9.67 (6.75, 13.86)
COVID-19 is not dangerous						
No	2,079 (47.66)	348 (7.97)	1,864 (42.73)	71 (1.62)	4,362 (95.55)	Ref.
Yes	181 (89.60)	21 (10.39)	0 (0)	0 (0)	202 (4.42)	10.35 (6.58, 16.28)
Fear of infection						
No	1,723 (42.87)	361 (8.98)	1,864 (46.37)	71 (1.76)	4,019 (88.04)	Ref.
Yes	537 (98.5)	8 (1.46)	0 (0)	0 (0)	545 (11.94)	90.67 (44.99, 182.73)
Against the principle of vaccination in general						
No	1,900 (46.14)	283 (6.87)	1,864 (45.26)	71 (1.72)	4,118 (90.21)	Ref.
Yes	360 (80.71)	86 (19.28)	0 (0)	0 (0)	446 (9.77)	5.96 (4.69, 7.57)
Religious reasons						
No	2,243 (49.40)	362 (7.97)	1,864 (41.05)	71 (1.56)	4,540 (99.45)	Ref.
Yes	17 (70.83)	7 (29.16)	0 (0)	0 (0)	24 (0.53)	3.29 (1.41, 7.65)
Traditional beliefs						
No	2,223 (49.10)	369 (8.15)	1,864 (41.17)	71 (1.56)	4,527 (99.17)	Ref.
Yes	37 (100)	0 (0)	0 (0)	0 (0)	37 (0.81)	34.17 (−8,474, 8,474)^b^
I believe in traditional and local medicine						
No	2,244 (49.35)	368 (8.09)	1,864 (40.99)	71 (1.56)	4,547 (99.61)	Ref.
Yes	16 (94.11)	1 (5.88)	0 (0)	0 (0)	17 (0.37)	17.18 (2.28, 129.19)
Other reasons						
No	1,878 (45.14)	347 (8.34)	1,864 (44.80)	71 (1.70)	4,160 (91.13)	Ref.
Yes	382 (94.55)	22 (5.44)	0 (0)	0 (0)	404 (8.85)	22.15 (14.36, 34.17)
Without reason						
No	2,259 (91.34)	214 (8.65)	0 (0)	0 (0)	2,473 (54.17)	Ref.
Yes	1 (0.04)	155 (7.41)	1,864 (89.14)	71 (3.39)	2091 (45.81)	0.00002 (0.00, 0.0001)

^a^ Based on univariate ordinal logistic regression.

^b^ Finding was reported as regression coefficient based on zero-inflated order logistic regression.

OR, odds ratio; CI, confidence interval; Ref., reference category.

Multivariate ordinal logistic regression showed that receiving at least one dose of COVID-19 vaccination is higher in age groups of 19–45 years (OR = 0.30; 95% CI: 0.20, 0.46), 46–65 years (OR = 0.19; 95% CI: 0.12, 0.29) and 65–98 years (OR = 0.22; 95% CI: 0.13, 0.38) than age less than 19 years of age. Women are less willing to prescribe the COVID-19 vaccine (OR = 1.25; 95% CI: 1.04, 1.50) and the Arab nationality have less tendency in receiving the COVID-19 vaccine (OR = 1.26; 95% CI: 1.02, 1.56). The analysis shows that less Sulaimani (OR = 3.04; 95% CI: 2.46, 3.75) and Ninawa (OR = 1.50; 95% CI: 1.19, 1.89) residents than other governates have gotten COVID-19 vaccines. It seems that there is an increasing trend in receiving the COVID-19 vaccine with increasing the level of education (Diploma or less: OR = 0.65; 95% CI: 0.52, 0.81 and university: OR = 0.40; 95% CI: 0.30, 0.54).

The results also indicated that there was an independent association between COVID-19 vaccination coverage and the possible attitudinal barriers of COVID-19 vaccination coverage. Fear of being unsafe (OR = 33.65; 95% CI: 25.97, 43.59), not being effective (OR = 25.05; 95% CI: 16.40, 38.27), COVID-19 is not dangerous (OR = 41.20; 95% CI: 24.71, 68.67), fear of infection following vaccination (OR = 431.15; 95% CI: 209.58, 886.94), inappropriate attitude against the principle of vaccination in general (OR = 27.16; 95% CI: 20.34, 36.28), religious reasons (OR = 9.81; 95% CI: 3.66, 26.28), believing on traditional and local medicine (OR = 56.55; 95% CI: 6.56, 487.24) and other reasons (OR = 87.35; 95% CI: 54.87, 139.06) were the important personal barriers against COVID-19 vaccination (Table 3).

**TABLE 3:** Multivariate ordered logistic regression to find independent risk factors against COVID-19 vaccination in all participants (Iraq April–May 2022).

Variable	aOR (95% CI)
Age group (year)	
12 to 19	Ref.
19 to 45	0.30 (0.20, 0.46)
46 to 65	0.19 (0.12, 0.29)
65 to 98	0.22 (0.13, 0.38)
Gender	
Male	Ref.
Female	1.25 (1.04, 1.50)
Nationality	
Kurd	Ref.
Arab	1.26 (1.02, 1.56)
Religion	
Muslim	Ref.
Christian	0.44 (0.23, 0.84)
Maritial status	
Married	Ref.
Divorced	0.61 (0.38, 0.99)
Governate	
Erbil	Ref.
Sulaimani	3.04 (2.46, 3.75)
Ninawa	1.50 (1.19, 1.89)
Education level	
Illiterate	Ref.
Diploma or less	0.65 (0.52, 0.81)
University	0.40 (0.30, 0.54)
Occupation	
Health and medical fields	Ref.
Office worker	1.51 (1.04, 2.21)
Non-office worker	2.32 (1.67, 3.24)
Student	2.90 (1.98, 4.23)
Retired	2.03 (1.20, 3.43)
Other	3.21 (2.40, 4.30)
Factors leading to avoid COVID-19 vaccination	
Unsafe	
No	Ref.
Yes	33.65 (25.97, 43.59)
Not effective	
No	Ref.
Yes	25.05 (16.40, 38.27)
Corona disease is not dangerous	
No	Ref.
Yes	41.20 (24.71, 68.67)
Fear of infection	
No	Ref.
Yes	431.15 (209.58, 886.94)
Against the principle of vaccination in general	
No	Ref.
Yes	27.16 (20.34, 36.28)
Religious reasons	
No	Ref.
Yes	9.81 (3.66, 26.28)
I believe in traditional and local medicine	
No	Ref.
Yes	56.55 (6.56, 487.24)
Other reasons	
No	Ref.
Yes	87.35 (54.87, 139.06)

aOR, adjusted odds ratio; CI, confidence interval.

### Sub-Population’s Influential Factors of COVID-19 Vaccination Coverage

#### Host Communities Influential Factors

Our results revealed that 51.49% of the participants of host communities had received at least one dose of COVID-19 vaccination. 42.28% of host communities subjects were vaccinated by two doses and 1.65% had received three doses of vaccination. Multivariate ordinal logistic regression showed that in host communities subjects, vaccination is higher in age groups of 19–45 years (OR = 0.22; 95% CI: 0.14, 0.36), 46–65 years (OR = 0.15; 95% CI: 0.09, 0.26) and 65–98 years (OR = 0.17; 95% CI: 0.09, 0.31) than age less than 19 years of age. Christians (OR = 0.42; 95% CI: 0.20, 0.90) and educated subjects (Diploma or less: OR = 0.53; 95% CI: 0.41, 0.68 and university: OR = 0.32; 95% CI: 0.23, 0.45) had more tendency in receiving COVID-19 vaccination. Beliefs of vaccination being unsafe (OR: 33.10; 95% CI: 24.48, 44.75), not effective (OR = 18.11; 95% CI: 11.41, 28.77), corona disease not being dangerous (OR = 42.42; 95% CI: 24.03, 74.87), fear of infection (OR = 339.24; 95% CI: 155.83, 738.54), being against the principle of vaccination in general (OR = 28.80; 95% CI: 20.45, 40.56) and religious reasons (OR = 11.14; 95% CI: 3.26, 38.10) were the personal attitude barriers against COVID-19 vaccination in host communities. Supplementary Tables S2–S4 present the baseline characteristics and risk factors against vaccination in host communities.

#### Internally Displaced Persons Influential Factors

Our results show that 48.83% of the IDPs had received at least one dose of vaccination, with 35.75% receiving two doses and 0.93% receiving three doses of COVID-19 vaccination. In multivariate analysis, IDPs were shown to have higher tendency for vaccination in age groups of 19–45 years (OR = 0.04; 95% CI: 0.01, 0.20), 46–65 years (OR = 0.02; 95% CI: 0.00, 0.10) and 65–98 years (OR = 0.01; 95% CI: 0.00, 0.03) than age less than 19 years of age and educated subjects (Diploma or less: OR = 0.50; 95% CI: 0.26, 0.96 and university: OR = 0.26; 95% CI: 0.10, 0.67) were also shown to have higher tendency for vaccination. However, females (OR = 2.36; 95% CI: 1.37, 4.07) and Arab (OR = 3.38; 95% CI: 1.56, 7.29) and Turkman (OR = 4.02; 95% CI: 1.65, 9.77) nationality subjects had lower tendency for vaccination. Believing that vaccination is unsafe (OR = 9.28; 95% CI: 4.71, 18.27), not effective (OR = 20.07; 95% CI: 3.98, 101.06) and being against principle of vaccination (OR = 16.19; 95% CI: 6.82, 38.43) were the personal attitude barriers against COVID-19 vaccination in IDPs. Supplementary Tables S5–S7 present the baseline characteristics and risk factors against vaccination in IDPs.

#### Refugees Influential Factors

Our results demonstrate that 45.87% of the refugee subjects received at least one dose of COVID-19 vaccination. 36.14% of refugee subjects received two doses and 1.46% received three doses of vaccination. Multivariate ordinal logistic regression revealed that not married (single) subjects had lower tendency for vaccination (OR = 3.53; 95% CI: 1.93, 6.46). As for the personal attitudinal barriers against COVID-19 vaccination in refugee subjects, belief in vaccination being unsafe (OR = 56.02; 95% CI: 26.83, 116.99), not being effective (OR = 303.28; 95% CI: 38.60, 2382.88), fear of infection (OR = 921.52; 95% CI: 121.76, 6974.44) and being against the principle of vaccination (OR = 15.32; 95% CI: 7.51, 31.27) were the most important factors against vaccination. Supplementary Tables S8–S10 present the baseline characteristics and risk factors against vaccination in refugee subjects.

## Discussion

Infectious diseases can be successfully controlled through vaccination; therefore, vaccination can be labeled as one of the most remarkable achievements of science. However, vaccination success can be negatively affected by people’s hesitation to get vaccinated. Therefore, there has always been the challenge of encouraging people’s desire to be vaccinated [16]. Iraq has experienced an alarming prevalence rate of COVID-19, with high daily reported new cases and hundreds of deaths reported monthly from June 2020. Therefore, combating COVID-19 in Iraq is only possible through vaccination [17]. However, vaccination hesitancy is still a big barrier to reaching high rates of vaccination in Iraq.

The results of the current study revealed that nearly half of the participants had not been vaccinated at all. In a study conducted on Iraqi population, Alatrany et al. reported that 68% of the study population had received atleast one vaccine dose; which is close to the results of our study [18], while in a report of vaccination rates in Duhok governorate of Iraq Kurdistan region, Abdulah revealed that 83.5% of the participants had not received a vaccine with 51.4% of them not intending to receive one [19]. This number falls short in comparison to neighboring countries such as Iran and Egypt in which almost 70 percent of the population have been reported to be fully vaccinated [7].

In their review Troiano and Nardi report that vaccination rate varies between the studies with a maximum of 77.6% of general population declaring that they will accept the COVID-19 vaccine [7] pointed out that vaccine acceptance can vary from community to community and from country to country, and this variation can be justified through various factors like the people’s trust in their government and national health organizations, education and public awareness, the economies of the countries, social and political conditions, and COVID-19 prevalence and mortality rates in the local community [20]. Over 40% of participants had been vaccinated by two doses and 1.56% received their third dose. In a similar study by Qin et al. (2022), it was reported that the rate of vaccine acceptance is higher among those who are willing to receive the third dose of vaccine. They also remarked that people in least developed countries are less likely to receive the third dose, which can be attributed to vaccination hesitancy [21].

The results of the current study showed that the participants’ place of residence, age group, gender, nationality, level of education, and occupation had a significant effect on COVID-19 vaccination coverage. Similarly, other studies indicated that willingness to receive COVID-19 vaccine varies in different communities and countries and is significantly influenced by factors like urban residence, being a physician or health professional, having children, previous interaction with someone infected by COVID-19, access to the media, and good practice of COVID-19 preventive measures [22, 23]. As suggested by the Health Cluster bulletin (2022), to achieve a broader coverage rate for COVID-19 vaccination, the epidemic indicators require continued emphasis by health partners on the importance of prevention, physical distancing, masking, and vaccination countrywide [13].

Regarding the reasons for avoiding vaccination in the present study, nearly 17% of the participants believed that the COVID-19 vaccine was not safe, 11.94% feared infection, and 10.79% feared its possible side effects. About 9.77% of them were generally against the principle of vaccination, and 6.46% said that vaccination could not be an effective option against COVID-19. In a study on the Iraqi population, Alatrany et al. [18] reported that distrust in government, social norms, perceived benefit of vaccination and severity of COVID-19 were significant predictors of vaccine hesitancy, while in contrast to our study, factors such as perceived infection likelihood and gender were not significant predictors of vaccine hesitancy. Abdulah has also investigated the vaccine hesitancy in Duhok governorate of Iraq Kurdistan region and reported that more than half the population are concerned about benefit of vaccines, their side-effects and new vaccine technologies. In his study it was demonstrated that education levels, occupation and concerns of adverse side-effects are significantly associated with the intention to vaccination while gender was not shown to have such association [19]. Tahir et al. have also investigated vaccine hesitancy in four governorates of Duhok, Erbil, Sulaiymaniy and Halabja of Iraq Kurdistan region and have reported that as much as 35% of the participants rejected to be vaccinated. Tahir et al demonstrated that age, occupation, higher level education and loosing a family member due to COVID-19 were significantly associated with vaccination intention [15]. In line with these findings, Mubarak et al (2022) reported that high acceptance of COVID-19 vaccine in university students in Saudi Arabia is determined by belief in the effectiveness and safety of the vaccine and trust in its capability to prevent the consequent complications, while fear of side effects is regarded a major factor for refusing vaccination [24].

Studies have reported varying degrees of COVID-19 vaccine hesitancy among refugees/migrants and asylum seekers, ranging between 10 and 40 percent [25]. Our results indicate that the reasons for avoiding vaccination was mostly similar between the host communities, IDPs and refugees which consisted of belief of vaccination being unsafe and not effective, fear of infection and being against the principle of vaccination in general. In a systematic review of vaccine acceptance and hesitancy among migrants and foreign workers, the potential barriers against vaccination were vaccine safety, mistrust of vaccines and healthcare system in general, newness of vaccines and low confidence in COVID-19 vaccines, assuming the disease is not dangerous, inadequate information, logistical barriers and religious prohibition [26].

Several interventions have been conducted in studies to increase the public’s willingness to receive the vaccine. A systematic review of 39 studies shows that communicating about vaccine concerns on social media does not reduce willingness to get vaccinated, but making vaccination mandatory has negative impact on vaccine uptake [27]. Governmental incentivization and persuasion are important factors to achieve higher vaccination coverage. Although, monetary incentives can increase the vaccination coverage [28], some believes incentives alone does not effective measures to encouraging vaccination [29]. It seems that financial incentivization do not enhanced COVID-19 vaccination in the vaccine hesitant [30]. Persuasion, prestige-based incentives, and adopting behaviorally informed policies are possible alternative means [29, 31, 32]. As a general recommendation, association of vaccine coverage with demographic, personal and geographical factors emphasize that a combination of social, cultural and even religious parameters should be considered to adopt effective measures to achieve proper vaccination coverage rate.

Our study is limited by no reports on the rate of refusal to participate in the study, which might lead to selection bias, not assessing the effect of the available vaccine type on participants vaccine hesitancy, and not investigating the accessibility of vaccination facilities, which might hinder vaccination in rural, deprived, or underprivileged districts. Future studies could address these issues in order to better investigate the contributing factors to vaccine hesitancy.

### Conclusion

Vaccination is one of the main acceptable options for preventing and controlling COVID-19; however, people’s refusal to accept the vaccine remains as a global challenge. It seems that due to such refusal, a very small portion of the participants in the present study received their third dose. Sociodemographic factors including age, gender, level of education, occupation, and nationality could significantly affect vaccination coverage. Moreover, higher acceptance rate of vaccination is associated with belief in vaccine safety and effectiveness and trust in the ability of the vaccine to prevent the complication. Hesitancy, uncertainty, and rumors regarding the vaccine should be minimized through the social media and appropriate health programs, resulting in controlling the pandemic through increasing the acceptance of COVID-19 vaccination.

## References

[1] WHO. COVID-19 Public Health Emergency of International Concern (PHEIC) Global Research and Innovation Forum (2020). Available From: https://www.who.int/publications/m/item/covid-19-public-health-emergency-of-international-concern-(pheic)-global-research-and-innovation-forum (Accessed June 21, 2022).

[2] Al-Khafaji ZA Abady NR Al-Kafaji HA. Epidemiological and Clinical Comparative Study for COVID-19 Patients in Babylon Province, Iraq. Arch Razi Inst (2022) 77(1):111–5. 10.22092/ARI.2021.356550.1869 35891720 PMC9288599

[3] Lafta RK Mawlood NA. Mental and Social Burden of COVID-19 on the Iraqi People. Int J Soc Psychiatry (2022) 69:200–7. 10.1177/00207640221077618 35176881 PMC9939619

[4] World Health Organization. Iraq: WHO Coronavirus Disease (COVID-19) Dashboard With Vaccination Data (2022). Available From: https://covid19.who.int/region/emro/country/iq (Accessed October 21, 2022).

[5] Lami F Rashak HA Khaleel HA Mahdi SG Adnan F Khader YS Iraq Experience in Handling the COVID-19 Pandemic: Implications of Public Health Challenges and Lessons Learned for Future Epidemic Preparedness Planning. J Public Health (2021) 43(3):iii19–iii28. 10.1093/pubmed/fdab369 PMC8660009 34651194

[6] Zheng C Shao W Chen X Zhang B Wang G Zhang W. Real-World Effectiveness of COVID-19 Vaccines: A Literature Review and Meta-Analysis. Int J Infect Dis (2022) 114:252–60. 10.1016/j.ijid.2021.11.009 34800687 PMC8595975

[7] World Health Organization. WHO Coronavirus Disease (COVID-19) Dashboard With Vaccination Dat (2023). Available From: https://covid19.who.int/?mapFilter=vaccinations (Accessed October 11, 2022).

[8] Troiano G Nardi A. Vaccine Hesitancy in the Era of COVID-19. Public Health (2021) 194:245–51. 10.1016/j.puhe.2021.02.025 33965796 PMC7931735

[9] Sherman SM Sim J Cutts M Dasch H Amlôt R Rubin GJ COVID-19 Vaccination Acceptability in the UK at the Start of the Vaccination Programme: A Nationally Representative Cross-Sectional Survey (CoVAccS–Wave 2). Public health (2022) 202:1–9. 10.1016/j.puhe.2021.10.008 34856520 PMC8520876

[10] Gallè F Sabella EA Roma P Da Molin G Diella G Montagna MT Acceptance of COVID-19 Vaccination in the Elderly: A Cross-Sectional Study in Southern Italy. Vaccines (2021) 9(11):1222. 10.3390/vaccines9111222 34835152 PMC8618111

[11] Bayou FD Amare SN. Acceptance of COVID-19 Vaccine and Its Associated Factors Among Ethiopian Population: A Systematic Review. Patient preference and adherence (2022) 16:1093–103. 10.2147/PPA.S360174 35492852 PMC9048957

[12] Solís Arce JS Warren SS Meriggi NF Scacco A McMurry N Voors M COVID-19 Vaccine Acceptance and Hesitancy in Low-and Middle-Income Countries. Nat Med (2021) 27(8):1385–94. 10.1038/s41591-021-01454-y 34272499 PMC8363502

[13] Reliefweb. Iraq: Health Cluster Bulletin No 5 (2022). Available From: https://reliefweb.int/report/iraq/iraq-health-cluster-bulletin-no-5-may-2022 (Accessed July 17, 2022).

[14] Henderson RH Sundaresan T. Cluster Sampling to Assess Immunization Coverage: A Review of Experience With a Simplified Sampling Method. Bull World Health Organ (1982) 60(2):253–60. 6980735 PMC2535957

[15] Tahir AI Ramadhan DS Piro SS Abdullah RY Taha AA Radha RH. COVID-19 Vaccine Acceptance, Hesitancy and Refusal Among Iraqi Kurdish Population. Int J Health Sci (Qassim) (2022) 16(1):10–6. 35024029 PMC8721214

[16] Greenwood B. The Contribution of Vaccination to Global Health: Past, Present and Future. Phil Trans R Soc Lond Ser B, Biol Sci (2014) 369(1645):20130433. 10.1098/rstb.2013.0433 24821919 PMC4024226

[17] Uctu R. Use of Generic Medicines in the Middle East: Knowledge, Perceptions and Experiences of the Sulaimani Population, KRI, Iraq. J Generic Medicines (2021) 17(4):206–13. 10.1177/17411343211008948

[18] Alatrany SSJ Falaiyah AM Zuhairawi RHM Ogden R Ali Sayyid Aldrraji H Alatrany ASS A Cross-Sectional Analysis of the Predictors of COVID-19 Vaccine Uptake and Vaccine Hesitancy in Iraq. PLoS One (2023) 18(3):e0282523. 10.1371/journal.pone.0282523 36893102 PMC9997880

[19] Abdulah DM. Prevalence and Correlates of COVID-19 Vaccine Hesitancy in the General Public in Iraqi Kurdistan: A Cross-Sectional Study. J Med Virol (2021) 93(12):6722–31. 10.1002/jmv.27255 34347294 PMC8427006

[20] Guidry JP Laestadius LI Vraga EK Miller CA Perrin PB Burton CW Willingness to Get the COVID-19 Vaccine With and Without Emergency Use Authorization. Am J Infect Control (2021) 49(2):137–42. 10.1016/j.ajic.2020.11.018 33227323 PMC7677682

[21] Qin C Wang R Tao L Liu M Liu J. Acceptance of a Third Dose of COVID-19 Vaccine and Associated Factors in China Based on Health Belief Model: A National Cross-Sectional Study. Vaccines (2022) 10(1):89. 10.3390/vaccines10010089 35062750 PMC8780099

[22] Mose A. Willingness to Receive COVID-19 Vaccine and Its Determinant Factors Among Lactating Mothers in Ethiopia: A Cross-Sectional Study. Infect Drug Resist (2021) 14:4249–59. 10.2147/IDR.S336486 34703251 PMC8523808

[23] Taye BT Amogne FK Demisse TL Zerihun MS Kitaw TM Tiguh AE Coronavirus Disease 2019 Vaccine Acceptance and Perceived Barriers Among university Students in Northeast Ethiopia: A Cross-Sectional Study. Clin Epidemiol Glob Health (2021) 12:100848. 10.1016/j.cegh.2021.100848 34395948 PMC8351076

[24] Mubarak AS Baabbad AS Almalki NA Alrbaiai GT Alsufyani GA Kabrah DK. Beliefs, Barriers, and Acceptance Associated With COVID-19 Vaccination Among Taif University Students in Saudi Arabia. J Fam Med Prim Care (2022) 11(1):224–32. 10.4103/jfmpc.jfmpc_1255_21 PMC8930104 35309633

[25] Nichol AA Parcharidi Z Al-Delaimy WK Kondilis E. Rapid Review of COVID-19 Vaccination Access and Acceptance for Global Refugee, Asylum Seeker and Undocumented Migrant Populations. Int J Public Health (2022) 67:1605508. 10.3389/ijph.2022.1605508 36618432 PMC9812946

[26] Hajissa K Mutiat HA Kaabi NA Alissa M Garout M Alenezy AA COVID-19 Vaccine Acceptance and Hesitancy Among Migrants, Refugees, and Foreign Workers: A Systematic Review and Meta-Analysis. Vaccines (2023) 11(6):1070. 10.3390/vaccines11061070 37376459 PMC10302060

[27] Batteux E Mills F Jones LF Symons C Weston D. The Effectiveness of Interventions for Increasing COVID-19 Vaccine Uptake: A Systematic Review. Vaccines (2022) 10(3):386. 10.3390/vaccines10030386 35335020 PMC8949230

[28] Campos-Mercade P Meier AN Schneider FH Meier S Pope D Wengström E. Monetary Incentives Increase COVID-19 Vaccinations. Science (2021) 374(6569):879–82. 10.1126/science.abm0475 34618594 PMC10765478

[29] Volpp KG Cannuscio CC. Incentives for Immunity—Strategies for Increasing COVID-19 Vaccine Uptake. New Engl J Med (2021) 385(1):e1. 10.1056/NEJMp2107719 34038633

[30] Chang T Jacobson M Shah M Pramanik R Shah SB. Financial Incentives and Other Nudges Do Not Increase Covid-19 Vaccinations Among the Vaccine Hesitant. United States: National Bureau of Economic Research (2021).

[31] Salali GD Uysal MS. COVID-19 Vaccine Hesitancy Is Associated With Beliefs on the Origin of the Novel Coronavirus in the UK and Turkey. Psychol Med (2021) 52:3750–2. 10.1017/S0033291720004067 PMC7609204 33070804

[32] Pennings S Symons X. Persuasion, Not Coercion or Incentivisation, Is the Best Means of Promoting COVID-19 Vaccination. J Med Ethics (2021) 47(10):709–11. 10.1136/medethics-2020-107076 33504627

