# Supplementary material for: COVID-19 Vaccination Among Diverse Population Groups in the Northern Governorates of Iraq
Source: Int J Public Health. 2023 Nov 28;68:1605736. doi: 10.3389/ijph.2023.1605736 (PMC10713705; doi:10.3389/ijph.2023.1605736)
Supplement: Supplementary file 1 [file Table8.docx]

Supplementary Table 8: Distribution of baseline characteristics of the refugee subjects according to number of COVID-19 vaccination doses

| **Variables** | **COVID-19 vaccination status** | | | | **Total (%)** |  | **OR* (95% CI)** |
| --- | --- | --- | --- | --- | --- | --- | --- |
|  | **No vaccination** | **One dose** | **Two doses** | **Three doses** |  |  |  |
| **Age group (year)** |  |  |  |  |  |  |  |
| 12 to 18 | 77 (70.64) | 12 (11.01) | 20 (18.35) | 0 (0.00) | 109 (17.67) |  | *Ref.* |
| 19 to 45 | 177 (52.84) | 23 (6.87) | 130 (38.81) | 5 (1.49) | 335 (54.29) |  | 0.42 (0.27, 0.67) |
| 46 to 65 | 62 (42.47) | 14 (9.59) | 66 (45.21) | 4 (2.74) | 146 (23.66) |  | 0.29 (0.17, 0.48) |
| 65 to 98 | 18 (66.67) | 2 (7.41) | 7 (25.93) | 0 (0.00) | 27 (4.38) |  | 0.79 (0.33, 1.91) |
| **Gender** |  |  |  |  |  |  |  |
| Male | 169 (50.75) | 27 (8.11) | 129 (38.74) | 8 (2.40) | 333 (53.97) |  | *Ref.* |
| Female | 165 (58.10) | 24 (8.45) | 94 (33.10) | 1 (0.35) | 284 (46.03) |  | 1.39 (1.02, 1.90) |
| **Governate** |  |  |  |  |  |  |  |
| Erbil | 130 (51.59) | 32 (12.70) | 83 (32.94) | 7 (2.78) | 252 (40.84) |  | *Ref.* |
| Sulaimani | 85 (81.73) | 5 (4.81) | 14 (13.46) | 0 (0.00) | 104 (16.86) |  | 4.02 (2.32, 6.97) |
| Duhok | 63 (48.84) | 5 (3.88) | 60 (46.51) | 1 (0.78) | 129 (20.91) |  | 0.79 (0.52, 1.19) |
| Kirkuk | 8 (34.78) | 4 (17.39) | 11 (47.83) | 0 (0.00) | 23 (3.73) |  | 0.61 (0.28, 1.33) |
| Ninawa | 48 (44.04) | 5 (4.59) | 55 (50.46) | 1 (0.92) | 109 (17.67) |  | 0.66 (0.42, 1.01) |
| **Nationality** |  |  |  |  |  |  |  |
| Kurd | 123 (50.62) | 27 (11.11) | 93 (38.27) | 0 (0.00) | 243 (39.38) |  | *Ref.* |
| Arab | 194 (56.23) | 21 (6.09) | 122 (35.36) | 8 (2.32) | 345 (55.92) |  | 1.11 (0.81, 1.53) |
| Assyrian | 6 (75.00) | 0 (0.00) | 2 (25.00) | 0 (0.00) | 8 (1.30) |  | 2.54 (0.50, 12.81) |
| Turkman | 9 (52.94) | 3 (17.65) | 5 (29.41) | 0 (0.00) | 17 (2.76) |  | 1.21 (0.48, 3.05) |
| Other | 2 (50.00) | 0 (0.00) | 1 (25.00) | 1 (25.00) | 4 (0.65) |  | 0.45 (0.05, 4.36) |
| **Religion** |  |  |  |  |  |  |  |
| Muslim | 285 (55.88) | 47 (9.22) | 169 (33.14) | 9 (1.76) | 510 (82.66) |  | *Ref.* |
| Yazedy | 37 (43.02) | 4 (4.65) | 45 (52.23) | 0 (0.00) | 86 (13.94) |  | 0.57 (0.36, 0.88) |
| Christian | 11 (55.00) | 0 (0.00) | 9 (45.00) | 0 (0.00) | 20 (3.24) |  | 0.84 (0.35, 2.05) |
| Other | 1 (100.00) | 0 (0.00) | 0 (0.00) | 0 (0.00) | 1 (0.16) |  | -** |
| **Marital status** |  |  |  |  |  |  |  |
| Married | 193 (48.61) | 33 (8.31) | 165 (41.56) | 6 (1.51) | 397 (64.34) |  | *Ref.* |
| Single | 127 (67.20) | 17 (8.99) | 42 (22.22) | 3 (1.59) | 189 (30.63) |  | 2.21 (1.55, 3.15) |
| Divorced | 13 (46.43) | 1 (3.57) | 14 (50.00) | 0 (0.00) | 28 (4.54) |  | 0.86 (0.41, 1.80) |
| Other | 1 (33.33) | 0 (0.00) | 2 (66.67) | 0 (0.00) | 3 (0.49) |  | 0.46 (0.05, 4.48) |
| **Education** |  |  |  |  |  |  |  |
| Illiterate | 98 (49.75) | 21 (10.66) | 76 (38.58) | 2 (1.02) | 197 (31.93) |  | *Ref.* |
| Diploma or less | 205 (57.42) | 28 (7.84) | 118 (33.05) | 6 (1.68) | 357 (57.86) |  | 1.28 (0.92, 1.80) |
| University | 31 (49.21) | 2 (3.17) | 29 (46.03) | 1 (1.59) | 63 (10.21) |  | 0.84 (0.49, 1.47) |
| **Occupation** |  |  |  |  |  |  |  |
| Health and medical fields | 1 (8.33) | 1 (8.33) | 10 (83.33) | 0 (0.00) | 12 (1.94) |  | *Ref.* |
| Office worker | 8 (44.44) | 1 (5.56) | 8 (44.44) | 1 (5.56) | 18 (2.92) |  | 3.60 (0.81, 16.01) |
| Non-office worker | 18 (39.13) | 3 (6.52) | 24 (52.17) | 1 (2.17) | 46 (7.46) |  | 3.17 (0.86, 11.68) |
| Military and security | 1 (5.26) | 4 (21.05) | 14 (73.68) | 0 (0.00) | 19 (3.08) |  | 1.30 (0.30, 5.62) |
| Student | 80 (66.67) | 14 (11.67) | 24 (20.00) | 2 (1.67) | 120 (19.45) |  | 10.88 (3.15, 37.58) |
| Retired | 7 (53.85) | 1 (7.69) | 5 (38.46) | 0 (0.00) | 13 (2.11) |  | 6.04 (1.24, 29.45) |
| Others | 219 (56.30) | 27 (6.94) | 138 (35.48) | 5 (1.29) | 389 (63.05) |  | 6.48 (1.96, 21.47) |
| **Health status** |  |  |  |  |  |  |  |
| Positive chronic disease | 59 (50.82) | 9 (7.76) | 45 (38.79) | 3 (2.59) | 116 (18.80) |  | *Ref.* |
| Healthy | 275 (54.89) | 42 (8.38) | 178 (35.53) | 6 (1.20) | 501 (81.20) |  | 1.21 (0.82, 1.79) |

*, Based on univariate ordinal logistic regression

**, OR could not be calculated due to frequency of categories with zero subjects.

OR: Odds ratio; CI: Confidence interval; Ref.: Reference category
