# Supplementary material for: COVID-19 Vaccination Among Diverse Population Groups in the Northern Governorates of Iraq
Source: Int J Public Health. 2023 Nov 28;68:1605736. doi: 10.3389/ijph.2023.1605736 (PMC10713705; doi:10.3389/ijph.2023.1605736)
Supplement: Supplementary file 2 [file Table7.docx]

Supplementary Table 7: Multivariate ordered logistic regression to for independent risk factors against COVID-19 vaccination in the internally displaced persons

| **Variable** | **aOR (95% CI)** |
| --- | --- |
| **Age group (year)** |  |
| 12 to 19 | *Ref.* |
| 19 to 45 | 0.04 (0.01, 0.20) |
| 46 to 65 | 0.02 (0.00, 0.10) |
| 65 to 98 | 0.01 (0.00, 0.03) |
| **Gender** |  |
| Male | *Ref.* |
| Female | 2.36 (1.37, 4.07) |
| **Nationality** |  |
| Kurd | *Ref.* |
| Arab | 3.38 (1.56, 7.29) |
| Turkman | 4.02 (1.65, 9.77) |
| **Governate** |  |
| Erbil | *Ref.* |
| Ninawa | 1.92 (0.88, 4.18) |
| **Education level** |  |
| Illiterate | *Ref.* |
| Diploma or less | 0.50 (0.26, 0.96) |
| University | 0.26 (0.10, 0.67) |
| **Occupation** |  |
| Health and medical fields | *Ref.* |
| Non-office worker | 2.71 (1.18, 6.23) |
| Retired | 94.65 (12.96, 690.99) |
| Other | 3.75 (1.75, 8.01) |
| **Factors leading to avoid COVID-19 vaccination** |  |
| Unsafe  No  Yes |  |
|  | *Ref.* |
|  | 9.28 (4.71, 18.27) |
| Not effective  No  Yes |  |
|  | *Ref.* |
|  | 20.07 (3.98, 101.06) |
| Against the principle of vaccination in general  No  Yes |  |
|  | *Ref.* |
|  | 16.19 (6.82, 38.43) |
| Other reasons  No  Yes |  |
|  | *Ref.* |
|  | 14.01 (4.17, 47.05) |

aOR: Adjusted odds ratio; CI: Confidence interval; Ref.: Reference category
