# Supplementary material for: COVID-19 Vaccination Among Diverse Population Groups in the Northern Governorates of Iraq
Source: Int J Public Health. 2023 Nov 28;68:1605736. doi: 10.3389/ijph.2023.1605736 (PMC10713705; doi:10.3389/ijph.2023.1605736)
Supplement: Supplementary file 3 [file Table9.docx]

Supplementary Table 9: Distribution of possible barriers of COVID-19 vaccination coverage in the refuge subjects according to number of doses

| **Variables** | **COVID-19 vaccination status** | | | | **Total (%)** | **OR* (95% CI)** |
| --- | --- | --- | --- | --- | --- | --- |
|  | **No vaccination** | **One dose** | **Two doses** | **Three doses** |  |  |
| **Side effects** | |  |  |  |  |  |
| No | 256 (47.50) | 51 (9.46) | 223 (41.37) | 9 (1.67) | 539 (87.36) | *Ref.* |
| Yes | 78 (100.00) | 0 (0.0) | 0 (0.00) | 0 (0.00) | 78 (12.64) | -** |
| **Unsafe** |  |  |  |  |  |  |
| No | 218 (44.58) | 39 (7.98) | 223 (45.60) | 9 (1.84) | 489 (79.25) | *Ref.* |
| Yes | 116 (90.63) | 12 (9.38) | 0 (0.00) | 0 (0.00) | 128 (20.75) | 13.39 (7.22, 24.84) |
| **Not effective** |  |  |  |  |  |  |
| No | 262 (48.16) | 50 (9.19) | 223 (40.99) | 9 (1.65) | 544 (88.17) | *Ref.* |
| Yes | 72 (98.63) | 1 (1.37) | 0 (0.00) | 0 (0.00) | 73 (11.83) | 78.44 (10.82, 568.44) |
| **COVID-19 is not dangerous** | | |  |  |  |  |
| No | 302 (51.62) | 51 (8.72) | 223 (38.12) | 9 (1.54) | 585 (94.81) | *Ref.* |
| Yes | 32 (100.00) | 0 (0.00) | 0 (0.00) | 0 (0.00) | 32 (5.19) | -** |
| **Fear of infection** |  |  |  |  |  |  |
| No | 233 (45.24) | 50 (9.71) | 223 (43.30) | 9 (1.75) | 515 (83.47) | *Ref.* |
| Yes | 101 (99.02) | 1 (0.98) | 0 (0.00) | 0 (0.00) | 102 (16.53) | 123.41 (17.10, 891.28) |
| **Against the principle of vaccination in general** | | | | | | |
| No | 309 (53.93) | 32 (5.58) | 223 (38.92) | 9 (1.57) | 573 (92.87) | *Ref.* |
| Yes | 25 (56.82) | 19 (43.18) | 0 (0.00) | 0 (0.00) | 44 (7.13) | 1.88 (1.07, 3.31) |
| **Religious reasons** |  |  |  |  |  |  |
| No | 334 (54.22) | 50 (8.12) | 223 (36.20) | 9 (1.46) | 616 (99.84) | *Ref.* |
| Yes | 0 (0.00) | 1 (100.00) | 0 (0.00) | 0 (0.00) | 1 (0.16) | 0.71 (0.04, 11.58) |
| **Traditional beliefs** |  |  |  |  |  |  |
| No | 325 (53.45) | 51 (8.39) | 223 (36.68) | 9 (1.48) | 608 (98.54) | *Ref.* |
| Yes | 9 (100.00) | 0 (0.00) | 0 (0.00) | 0 (0.00) | 9 (1.46) | -** |
| **I believe in traditional and local medicine** | | | | | | |
| No | 333 (54.15) | 50 (8.13) | 223 (36.26) | 9 (1.46) | 615 (99.68) | *Ref.* |
| Yes | 1 (50.00) | 1 (50.00) | 0 (0.00) | 0 (0.00) | 2 (0.32) | 1.52 (0.14, 16.98) |
| **Other reasons** |  |  |  |  |  |  |
| No | 291 (50.87) | 49 (8.57) | 223 (38.99) | 9 (1.57) | 572 (92.71) | *Ref.* |
| Yes | 43 (95.56) | 2 (4.44) | 0 (0.00) | 0 (0.00) | 45 (7.29) | 21.55 (5.18, 89.68) |
| **Without reason** |  |  |  |  |  |  |
| No | 334 (90.76) | 34 (9.24) | 0 (0.00) | 0 (0.00) | 368 (59.64) | *Ref.* |
| Yes | 0 (0.00) | 17 (6.83) | 223 (89.56) | 9 (3.61) | 249 (40.36) | -** |

*, Based on univariate ordinal logistic regression

**, OR could not be calculated due to frequency of categories with zero subjects.

OR: Odds ratio; CI: Confidence interval; Ref.: Reference category
