# Supplementary material for: COVID-19 Vaccination Among Diverse Population Groups in the Northern Governorates of Iraq
Source: Int J Public Health. 2023 Nov 28;68:1605736. doi: 10.3389/ijph.2023.1605736 (PMC10713705; doi:10.3389/ijph.2023.1605736)
Supplement: Supplementary file 4 [file Table2.docx]

**Supplementary Material 2.**

Supplementary Table 2: Distribution of baseline characteristics of the host community’s subjects according to number of COVID-19 vaccination doses

| **Variables** | **COVID-19 vaccination status** | | | | **Total (%)** |  | **OR* (95% CI)** |
| --- | --- | --- | --- | --- | --- | --- | --- |
|  | **No vaccination** | **One dose** | **Two doses** | **Three doses** |  |  |  |
| **Age group (year)** |  |  |  |  |  |  |  |
| 12 to 18 | 268 (81.96) | 19 (5.81) | 40 (12.23) | 0 (0.00) | 327 (9.29) |  | *Ref.* |
| 19 to 45 | 978 (46.82) | 186 (8.90) | 899 (43.03) | 26 (1.24) | 2089 (59.36) |  | 0.19 (0.14, 0.26) |
| 46 to 65 | 330 (39.47) | 51 (6.10) | 429 (51.32) | 26 (3.11) | 836 (23.76) |  | 0.13 (0.10, 0.18) |
| 65 to 98 | 131 (49.06) | 10 (3.75) | 120 (44.94) | 6 (2.25) | 267 (7.59) |  | 0.19 (0.13, 0.27) |
| **Gender** |  |  |  |  |  |  |  |
| Male | 798 (41.74) | 157 (8.21) | 916 947.91) | 41 (2.14) | 1912 (54.33) |  | *Ref.* |
| Female | 909 (56.57) | 109 (6.78) | 572 (35.59) | 17 (1.06) | 1607 (45.67) |  | 1.78 (1.57, 2.03) |
| **Governate** |  |  |  |  |  |  |  |
| Erbil | 468 (47.66) | 87 (8.86) | 404 (41.14) | 23 (2.34) | 982 (27.91) |  | *Ref.* |
| Sulaimani | 625 (63.32) | 41 (4.15) | 317 (32.12) | 4 (0.41) | 987 (28.05) |  | 1.82 (1.52, 2.22) |
| Duhok | 88 (31.10) | 14 (4.95) | 175 (61.84) | 6 (2.12) | 283 (8.04) |  | 0.48 (0.37, 0.62) |
| Kirkuk | 119 (30.28) | 30 (7.63) | 240 (61.07) | 4 (1.02) | 393 (11.17) |  | 0.51 (0.40, 0.63) |
| Ninawa | 407 (46.57) | 94 (10.76) | 352 (40.27) | 21 (2.40) | 874 (24.84) |  | 1.00 (0.83, 1.18) |
| **Nationality** |  |  |  |  |  |  |  |
| Kurd | 1232 (51.63) | 162 (6.79) | 954 (39.98) | 38 (1.59) | 2386 (67.80) |  | *Ref.* |
| Arab | 430 (42.83) | 95 (9.46) | 463 (46.12) | 16 (1.59) | 1004 (28.53) |  | 0.75 (0.65, 0.86) |
| Assyrian | 10 (28.57) | 2 (5.71) | 21 (60.00) | 2 (5.71) | 35 (0.99) |  | 0.35 (0.18, 0.70) |
| Turkman | 31 (36.05) | 7 (8.14) | 46 (53.49) | 2 (2.33) | 86 (2.44) |  | 0.55 (0.36, 0.83) |
| Other | 4 (50.00) | 0 (0.00) | 4 (50.00) | 0 (0.00) | 8 (0.23) |  | 0.84 (0.21, 3.32) |
| **Religion** |  |  |  |  |  |  |  |
| Muslim | 1666 (48.63) | 260 (7.59) | 1447 (42.24) | 53 (1.55) | 3426 (97.36) |  | *Ref.* |
| Yazedy | 20 (55.56) | 3 (8.33) | 13 (36.11) | 0 (0.00) | 36 (1.02) |  | 1.36 (0.72, 2.58) |
| Christian | 21 (38.18) | 3 (5.45) | 26 (47.27) | 5 (9.09) | 55 (1.56) |  | 0.54 (0.31, 0.92) |
| Other | 0 (0.00) | 0 (0.00) | 2 (100.00) | 0 (0.00) | 2 (0.06) |  | 0.11 (0.01, 2.15) |
| **Marital status** |  |  |  |  |  |  |  |
| Married | 1097 (44.56) | 176 (7.15) | 1139 (46.26) | 50 (2.03) | 2462 (69.96) |  | *Ref.* |
| Single | 545 (58.41) | 80 (8.57) | 304 (32.58) | 4 (0.43) | 933 (26.51) |  | 1.82 (1.57, 2.11) |
| Divorced | 64 (56.15) | 5 (4.39) | 41 (35.96) | 4 (3.51) | 114 (3.24) |  | 1.48 (1.02, 2.15) |
| Other | 1 (10.00) | 5 (50.00) | 4 (40.00) | 0 (0.00) | 10 (0.28) |  | 0.72 (0.26, 1.99) |
| **Education** |  |  |  |  |  |  |  |
| Illiterate | 466 (61.64) | 52 (6.88) | 236 (31.22) | 2 (0.26) | 756 (21.48) |  | *Ref.* |
| Diploma or less | 997 (53.69) | 132 (7.11) | 708 (38.13) | 20 (1.08) | 1857 (52.77) |  | 0.71 (0.60, 0.84) |
| University | 244 (26.93) | 82 (9.05) | 544 (60.04) | 36 (3.97) | 906 (25.75) |  | 0.24 (0.20, 0.29) |
| **Occupation** |  |  |  |  |  |  |  |
| Health and medical fields | 32 (12.85) | 7 (2.81) | 191 (76.71) | 19 (7.63) | 249 (7.08) |  | *Ref.* |
| Office worker | 88 (27.08) | 23 (7.08) | 203 (62.46) | 11 (3.38) | 325 (9.24) |  | 2.80 (1.92, 4.09) |
| Non-office worker | 185 (41.57) | 60 (13.48) | 194 (43.60) | 6 (1.35) | 445 (12.65) |  | 6.12 (4.30, 8.72) |
| Military and security | 28 (13.59) | 11 (5.34) | 165 (80.10) | 2 (0.97) | 206 (5.85) |  | 1.54 (1.01, 2.34) |
| Student | 333 (63.67) | 55 (10.52) | 135 (25.81) | 0 (0.00) | 523 (14.86) |  | 14.35 (10.06, 20.48) |
| Retired | 53 (35.81) | 5 (3.38) | 83 (56.08) | 7 (4.73) | 148 (4.21) |  | 3.57 (2.28, 5.60) |
| Others | 988 (60.87) | 105 (6.47) | 517 (31.85) | 13 (0.80) | 1623 (46.12) |  | 11.84 (8.55, 16.38) |
| **Health status** |  |  |  |  |  |  |  |
| Positive chronic disease | 374 (47.58) | 51 (6.49) | 338 (43.00) | 23 (2.93) | 786 (22.34) |  | *Ref.* |
| Healthy | 1333 (48.77) | 215 (7.87) | 1150 (42.08) | 35 (1.28) | 2733 (77.66) |  | 1.11 (0.95, 1.29) |

*, Based on univariate ordinal logistic regression

OR: Odds ratio; CI: Confidence interval; Ref.: Reference category
