# Supplementary material for: COVID-19 Vaccination Among Diverse Population Groups in the Northern Governorates of Iraq
Source: Int J Public Health. 2023 Nov 28;68:1605736. doi: 10.3389/ijph.2023.1605736 (PMC10713705; doi:10.3389/ijph.2023.1605736)
Supplement: Supplementary file 5 [file Table5.docx]

Supplementary Table 5: Distribution of baseline characteristics of internally displaced persons according to number of COVID-19 vaccination doses

| **Variables** | **COVID-19 vaccination status** | | | | **Total (%)** |  | **OR* (95% CI)** |
| --- | --- | --- | --- | --- | --- | --- | --- |
|  | **No vaccination** | **One dose** | **Two doses** | **Three doses** |  |  |  |
| **Age group (year)** |  |  |  |  |  |  |  |
| 12 to 18 | 18 (81.82) | 1 (4.55) | 3 (13.64) | 0 (0.00) | 22 (5.14) |  | *Ref.* |
| 19 to 45 | 149 (50.68) | 35 (11.90) | 108 (36.73) | 2 (0.68) | 294 (68.69) |  | 0.23 (0.08, 0.70) |
| 46 to 65 | 46 (48.42) | 15 (15.79) | 32 (33.68) | 2 (2.11) | 95 (22.20) |  | 0.22 (0.07, 0.70) |
| 65 to 98 | 6 (35.29) | 1 (5.88) | 10 (58.82) | 0 (0.00) | 17 (3.97) |  | 0.11 (0.03, 0.46) |
| **Gender** |  |  |  |  |  |  |  |
| Male | 105 (43.21) | 33 (13.58) | 101 (41.56) | 4 (1.65) | 243 (56.78) |  | *Ref.* |
| Female | 114 (61.62) | 19 (10.27) | 52 (28.11) | 0 (0.00) | 185 (43.22) |  | 2.07 (1.42, 3.02) |
| **Governate** |  |  |  |  |  |  |  |
| Erbil | 2 (12.50) | 3 (18.75) | 11 (68.75) | 0 (0.00) | 16 (3.74) |  | *Ref.* |
| Sulaimani | 11 (44.00) | 3 (12.00) | 11 (44.00) | 0 (0.00) | 25 (5.84) |  | 3.03 (0.89, 10.33) |
| Duhok | 8 (17.78) | 3 (6.67) | 34 (75.56) | 0 (0.00) | 45 (10.51) |  | 0.85 (0.27, 2.70) |
| Kirkuk | 6 (30.00) | 4 (20.00) | 8 (40.00) | 2 (10.00) | 20 (4.67) |  | 1.70 (0.46, 6.23) |
| Ninawa | 192 (59.63) | 39 (12.11) | 89 (27.64) | 2 (0.62) | 322 (75.23) |  | 5.80 (2.13, 15.76) |
| **Nationality** |  |  |  |  |  |  |  |
| Kurd | 29 (29.00) | 9 (9.00) | 59 (59.00) | 3 (3.00) | 100 (23.36) |  | *Ref.* |
| Arab | 95 (51.91) | 30 (16.39) | 58 (31.69) | 0 (0.00) | 183 (42.76) |  | 3.20 (1.97, 5.19) |
| Assyrian | 0 (0.00) | 1 (16.67) | 5 (83.33) | 0 (0.00 | 6 (1.40) |  | 0.37 (0.06, 2.24) |
| Turkman | 95 (69.34) | 12 (8.76) | 29 (21.17) | 1 (0.73) | 137 (32.01) |  | 6.16 (3.59, 10.56) |
| Other | 0 (0.00) | 0 (0.00) | 2 (100.00) | 0 (0.00) | 2 (0.47) |  | 0.18 (0.01, 4.92) |
| **Religion** |  |  |  |  |  |  |  |
| Muslim | 219 (52.02) | 51 (12.11) | 147 (34.92) | 4 (0.95) | 421 (98.36) |  | *Ref.* |
| Yazedy | 0 (0.00) | 0 (0.00) | 0 (0.00) | 0 (0.00) | 0 (0.00) |  | -- |
| Christian | 0 (0.00) | 1 (14.29) | 6 (85.71) | 0 (0.00) | 7 (1.64) |  | 0.12 (0.02, 0.63) |
| Other | 0 (0.00) | 0 (0.00) | 0 (0.00) | 0 (0.00) | 0 (0.00) |  | -- |
| **Marital status** |  |  |  |  |  |  |  |
| Married | 167 (50.45) | 45 (13.60) | 115 (34.74) | 4 (1.21) | 331 (77.34) |  | *Ref.* |
| Single | 43 (55.84) | 5 (6.49) | 29 (37.66) | 0 (0.00) | 77 (17.99) |  | 1.12 (0.70, 1.83) |
| Divorced | 8 (47.06) | 1 (5.88) | 8 (47.06) | 0 (0.00) | 17 (3.97) |  | 0.77 (0.30, 1.97) |
| Other | 1 (33.33) | 1 (33.33) | 1 (33.33) | 0 (0.00) | 3 (0.70) |  | 0.76 (0.10, 5.46) |
| **Education** |  |  |  |  |  |  |  |
| Illiterate | 71 (68.93) | 7 (6.80) | 24 (23.30) | 1 (0.97) | 103 (24.07) |  | *Ref.* |
| Diploma or less | 135 (52.94) | 36 (14.12) | 82 (32.16) | 2 (0.78) | 255 (59.58) |  | 0.54 (0.34, 0.88) |
| University | 13 (18.57) | 9 (12.86) | 47 (67.14) | 1 (1.43) | 70 (16.36) |  | 0.13 (0.07, 0.24) |
| **Occupation** |  |  |  |  |  |  |  |
| Health and medical fields | 3 (8.11) | 4 (10.81) | 30 (81.08) | 0 (0.00) | 37 (8.64) |  | *Ref.* |
| Office worker | 7 (26.92) | 6 (23.08) | 12 (46.15) | 1 (3.85) | 26 (6.07) |  | 2.98 (1.08, 8.23) |
| Non-office worker | 36 (46.75) | 16 (20.78) | 23 (29.87) | 2 (2.60) | 77 (17.99) |  | 6.25 (2.71, 14.39) |
| Military and security | 6 (27.27) | 2 (9.09) | 14 (63.64) | 0 (0.00) | 22 (5.14) |  | 2.29 (0.77, 6.84) |
| Student | 16 (53.33) | 1 (3.33) | 13 (43.33) | 0 (0.00) | 30 (7.01) |  | 6.17 (2.24, 16.99) |
| Retired | 5 (71.43) | 1 (14.29) | 1 (14.29) | 0 (0.00) | 7 (1.64) |  | 17.10 (2.93, 99.87) |
| Others | 146 (63.76) | 22 (9.61) | 60 (26.20) | 1 (0.44) | 229 (53.50) |  | 10.97 (5.07, 23.76) |
| **Health status** |  |  |  |  |  |  |  |
| Positive chronic disease | 43 (44.79) | 13 (13.54) | 37 (38.54) | 3 (3.13) | 96 (22.43) |  | *Ref.* |
| Healthy | 176 (53.01) | 39 (11.75) | 116 (34.94) | 1 (0.30) | 332 (77.57) |  | 1.41 (0.91, 2.17) |

*, Based on univariate ordinal logistic regression

OR: Odds ratio; CI: Confidence interval; Ref.: Reference category
