# Supplementary material for: COVID-19 Vaccination Among Diverse Population Groups in the Northern Governorates of Iraq
Source: Int J Public Health. 2023 Nov 28;68:1605736. doi: 10.3389/ijph.2023.1605736 (PMC10713705; doi:10.3389/ijph.2023.1605736)
Supplement: Supplementary file 6 [file Table3.docx]

Supplementary Table 3: Distribution of possible barriers of COVID-19 vaccination coverage in the host communities according to number of doses

| **Variables** | **COVID-19 vaccination status** | | | | **Total (%)** | **OR* (95% CI)** |
| --- | --- | --- | --- | --- | --- | --- |
|  | **No vaccination** | **One dose** | **Two doses** | **Three doses** |  |  |
| **Side effects** | |  |  |  |  |  |
| No | 1349 (42.68) | 266 (8.42) | 1488 (47.07) | 58 (1.83) | 3161 (89.83) | *Ref.* |
| Yes | 358 (100) | 0 (0.00) | 0 (0.00) | 0 (0.00) | 358 (10.17) | -** |
| **Unsafe** |  |  |  |  |  |  |
| No | 1222 (41.31) | 190 (6.42) | 1488 (50.30) | 58 (1.96) | 2958 (84.06) | *Ref.* |
| Yes | 485 (86.45) | 76 (13.55) | 0 (0.00) | 0 (0.00) | 561 (15.94) | 10.59 (8.25, 13.61) |
| **Not effective** |  |  |  |  |  |  |
| No | 1540 (46.37) | 235 (7.08) | 1488 (44.81) | 58 (1.75) | 3321 (94.37) | *Ref.* |
| Yes | 167 (84.34) | 31 (15.66) | 0 (0.00) | 0 (0.00) | 198 (5.63) | 7.22 (4.91, 10.60) |
| **COVID-19 is not dangerous** | |  |  |  |  |  |
| No | 1560 (46.50) | 249 (7.42) | 1488 (44.35) | 58 (1.73) | 3355 (95.34) | *Ref.* |
| Yes | 147 (89.63) | 17 (10.37) | 0 (0.00) | 0 (0.00) | 164 (4.66) | 10.90 (6.58, 18.05) |
| **Fear of infection** |  |  |  |  |  |  |
| No | 1325 (42.33) | 259 (8.27) | 1488 (47.54) | 58 (1.85) | 3130 (88.95) | *Ref.* |
| Yes | 382 (98.20) | 7 (1.80) | 0 (0.00) | 0 (0.00) | 389 (11.05) | 75.60 (35.68, 160.17) |
| **Against the principle of vaccination in general** | | | | | | |
| No | 1427 (44.86) | 208 (6.54) | 1488 (46.78) | 58 (1.82) | 3181 (90.39) | *Ref.* |
| Yes | 280 (82.84) | 58 (17.16) | 0 (0.00) | 0 (0.00) | 338 (9.61) | 7.08 (5.31, 9.44) |
| **Religious reasons** |  |  |  |  |  |  |
| No | 1691 (48.33) | 262 (7.49) | 1488 (42.53) | 58 (1.66) | 3499 (99.43) | *Ref.* |
| Yes | 16 (80.00) | 4 (20.00) | 0 (0.00) | 0 (0.00) | 20 (0.57) | 5.11 (1.74, 15.00) |
| **Traditional beliefs** |  |  |  |  |  |  |
| No | 1687 (48.21) | 266 (7.60) | 1488 (42.53) | 58 (1.66) | 3499 (99.43) | *Ref.* |
| Yes | 20 (100.00) | 0 (0.00) | 0 (0.00) | 0 (0.00) | 20 (0.57) | -** |
| **I believe in traditional and local medicine** | | | | | | |
| No | 1693 (48.30) | 266 (7.59) | 1488 (42.45) | 58 (1.65) | 3505 (99.60) | *Ref.* |
| Yes | 14 (100.00) | 0 (0.00) | 0 (0.00) | 0 (0.00) | 14 (0.40) | -** |
| **Other reasons** |  |  |  |  |  |  |
| No | 1390 (43.63) | 250 (7.85) | 1488 (46.70) | 58 (1.82) | 3186 (90.54) | *Ref.* |
| Yes | 317 (95.20) | 16 (4.80) | 0 (0.00) | 0 (0.00) | 333 (9.46) | 26.76 (16.13, 44.41) |
| **Without reason** |  |  |  |  |  |  |
| No | 1706 (91.67) | 155 (8.33) | 0 (0.00) | 0 (0.00) | 1861 (52.88) | *Ref.* |
| Yes | 1 (0.06) | 111 (6.69) | 1488 (89.75) | 58 (3.50) | 1658 (47.12) | 0.00002 (0.00, 0.0001) |

*, Based on univariate ordinal logistic regression

**, OR could not be calculated due to frequency of categories with zero subjects.

OR: Odds ratio; CI: Confidence interval; Ref.: Reference category

Supplementary Table 4: Multivariate ordered logistic regression to for independent risk factors against COVID-19 vaccination in the host communities

| **Variable** | **aOR (95% CI)** |
| --- | --- |
| **Age group (year)** |  |
| 12 to 19 | *Ref.* |
| 19 to 45 | 0.22 (0.14, 0.36) |
| 46 to 65 | 0.15 (0.09, 0.26) |
| 65 to 98 | 0.17 (0.09, 0.31) |
| **Religion** |  |
| Muslim | *Ref.* |
| Christian | 0.42 (0.20, 0.90) |
| **Governate** |  |
| Erbil | *Ref.* |
| Sulaimani | 2.68 (2.14, 3.37) |
| Ninawa | 1.86 (1.47, 2.36) |
| **Education level** |  |
| Illiterate | *Ref.* |
| Diploma or less | 0.53 (0.41, 0.68) |
| University | 0.32 (0.23, 0.45) |
| **Occupation** |  |
| Health and medical fields | *Ref.* |
| Office worker | 1.43 (0.97, 2.13) |
| Non-office worker | 2.13 (1.51, 3.02) |
| Student | 2.95 (1.97, 4.43) |
| Other | 3.32 (2.50, 4.41) |
| **Factors leading to avoid COVID-19 vaccination** |  |
| Unsafe  No  Yes |  |
|  | *Ref.* |
|  | 33.10 (24.48, 44.75) |
| Not effective  No  Yes |  |
|  | *Ref.* |
|  | 18.11 (11.41, 28.77) |
| Corona disease is not dangerous  No  Yes |  |
|  | *Ref.* |
|  | 42.42 (24.03, 74.87) |
| Fear of infection  No  Yes |  |
|  | *Ref.* |
|  | 339.24 (155.83, 738.54) |
| Against the principle of vaccination in general  No  Yes |  |
|  | *Ref.* |
|  | 28.80 (20.45, 40.56) |
| Religious reasons  No  Yes |  |
|  | *Ref.* |
|  | 11.14 (3.26, 38.10) |
| Other reasons  No  Yes |  |
|  | *Ref.* |
|  | 103.78 (60.07, 179.27) |

aOR: Adjusted odds ratio; CI: Confidence interval

Supplementary Table 5: Distribution of baseline characteristics of internally displaced persons according to number of COVID-19 vaccination doses

| **Variables** | **COVID-19 vaccination status** | | | | **Total (%)** |  | **OR* (95% CI)** |
| --- | --- | --- | --- | --- | --- | --- | --- |
|  | **No vaccination** | **One dose** | **Two doses** | **Three doses** |  |  |  |
| **Age group (year)** |  |  |  |  |  |  |  |
| 12 to 18 | 18 (81.82) | 1 (4.55) | 3 (13.64) | 0 (0.00) | 22 (5.14) |  | *Ref.* |
| 19 to 45 | 149 (50.68) | 35 (11.90) | 108 (36.73) | 2 (0.68) | 294 (68.69) |  | 0.23 (0.08, 0.70) |
| 46 to 65 | 46 (48.42) | 15 (15.79) | 32 (33.68) | 2 (2.11) | 95 (22.20) |  | 0.22 (0.07, 0.70) |
| 65 to 98 | 6 (35.29) | 1 (5.88) | 10 (58.82) | 0 (0.00) | 17 (3.97) |  | 0.11 (0.03, 0.46) |
| **Gender** |  |  |  |  |  |  |  |
| Male | 105 (43.21) | 33 (13.58) | 101 (41.56) | 4 (1.65) | 243 (56.78) |  | *Ref.* |
| Female | 114 (61.62) | 19 (10.27) | 52 (28.11) | 0 (0.00) | 185 (43.22) |  | 2.07 (1.42, 3.02) |
| **Governate** |  |  |  |  |  |  |  |
| Erbil | 2 (12.50) | 3 (18.75) | 11 (68.75) | 0 (0.00) | 16 (3.74) |  | *Ref.* |
| Sulaimani | 11 (44.00) | 3 (12.00) | 11 (44.00) | 0 (0.00) | 25 (5.84) |  | 3.03 (0.89, 10.33) |
| Duhok | 8 (17.78) | 3 (6.67) | 34 (75.56) | 0 (0.00) | 45 (10.51) |  | 0.85 (0.27, 2.70) |
| Kirkuk | 6 (30.00) | 4 (20.00) | 8 (40.00) | 2 (10.00) | 20 (4.67) |  | 1.70 (0.46, 6.23) |
| Ninawa | 192 (59.63) | 39 (12.11) | 89 (27.64) | 2 (0.62) | 322 (75.23) |  | 5.80 (2.13, 15.76) |
| **Nationality** |  |  |  |  |  |  |  |
| Kurd | 29 (29.00) | 9 (9.00) | 59 (59.00) | 3 (3.00) | 100 (23.36) |  | *Ref.* |
| Arab | 95 (51.91) | 30 (16.39) | 58 (31.69) | 0 (0.00) | 183 (42.76) |  | 3.20 (1.97, 5.19) |
| Assyrian | 0 (0.00) | 1 (16.67) | 5 (83.33) | 0 (0.00 | 6 (1.40) |  | 0.37 (0.06, 2.24) |
| Turkman | 95 (69.34) | 12 (8.76) | 29 (21.17) | 1 (0.73) | 137 (32.01) |  | 6.16 (3.59, 10.56) |
| Other | 0 (0.00) | 0 (0.00) | 2 (100.00) | 0 (0.00) | 2 (0.47) |  | 0.18 (0.01, 4.92) |
| **Religion** |  |  |  |  |  |  |  |
| Muslim | 219 (52.02) | 51 (12.11) | 147 (34.92) | 4 (0.95) | 421 (98.36) |  | *Ref.* |
| Yazedy | 0 (0.00) | 0 (0.00) | 0 (0.00) | 0 (0.00) | 0 (0.00) |  | -- |
| Christian | 0 (0.00) | 1 (14.29) | 6 (85.71) | 0 (0.00) | 7 (1.64) |  | 0.12 (0.02, 0.63) |
| Other | 0 (0.00) | 0 (0.00) | 0 (0.00) | 0 (0.00) | 0 (0.00) |  | -- |
| **Marital status** |  |  |  |  |  |  |  |
| Married | 167 (50.45) | 45 (13.60) | 115 (34.74) | 4 (1.21) | 331 (77.34) |  | *Ref.* |
| Single | 43 (55.84) | 5 (6.49) | 29 (37.66) | 0 (0.00) | 77 (17.99) |  | 1.12 (0.70, 1.83) |
| Divorced | 8 (47.06) | 1 (5.88) | 8 (47.06) | 0 (0.00) | 17 (3.97) |  | 0.77 (0.30, 1.97) |
| Other | 1 (33.33) | 1 (33.33) | 1 (33.33) | 0 (0.00) | 3 (0.70) |  | 0.76 (0.10, 5.46) |
| **Education** |  |  |  |  |  |  |  |
| Illiterate | 71 (68.93) | 7 (6.80) | 24 (23.30) | 1 (0.97) | 103 (24.07) |  | *Ref.* |
| Diploma or less | 135 (52.94) | 36 (14.12) | 82 (32.16) | 2 (0.78) | 255 (59.58) |  | 0.54 (0.34, 0.88) |
| University | 13 (18.57) | 9 (12.86) | 47 (67.14) | 1 (1.43) | 70 (16.36) |  | 0.13 (0.07, 0.24) |
| **Occupation** |  |  |  |  |  |  |  |
| Health and medical fields | 3 (8.11) | 4 (10.81) | 30 (81.08) | 0 (0.00) | 37 (8.64) |  | *Ref.* |
| Office worker | 7 (26.92) | 6 (23.08) | 12 (46.15) | 1 (3.85) | 26 (6.07) |  | 2.98 (1.08, 8.23) |
| Non-office worker | 36 (46.75) | 16 (20.78) | 23 (29.87) | 2 (2.60) | 77 (17.99) |  | 6.25 (2.71, 14.39) |
| Military and security | 6 (27.27) | 2 (9.09) | 14 (63.64) | 0 (0.00) | 22 (5.14) |  | 2.29 (0.77, 6.84) |
| Student | 16 (53.33) | 1 (3.33) | 13 (43.33) | 0 (0.00) | 30 (7.01) |  | 6.17 (2.24, 16.99) |
| Retired | 5 (71.43) | 1 (14.29) | 1 (14.29) | 0 (0.00) | 7 (1.64) |  | 17.10 (2.93, 99.87) |
| Others | 146 (63.76) | 22 (9.61) | 60 (26.20) | 1 (0.44) | 229 (53.50) |  | 10.97 (5.07, 23.76) |
| **Health status** |  |  |  |  |  |  |  |
| Positive chronic disease | 43 (44.79) | 13 (13.54) | 37 (38.54) | 3 (3.13) | 96 (22.43) |  | *Ref.* |
| Healthy | 176 (53.01) | 39 (11.75) | 116 (34.94) | 1 (0.30) | 332 (77.57) |  | 1.41 (0.91, 2.17) |

*, Based on univariate ordinal logistic regression

OR: Odds ratio; CI: Confidence interval; Ref.: Reference category

Supplementary Table 6: Distribution of possible barriers of COVID-19 vaccination coverage in the internally displaced persons according to number of doses

| **Variables** | **COVID-19 vaccination status** | | | | **Total (%)** | **OR* (95% CI)** |
| --- | --- | --- | --- | --- | --- | --- |
|  | **No vaccination** | **One dose** | **Two doses** | **Three doses** |  |  |
| **Side effects** | |  |  |  |  |  |
| No | 166 (44.27) | 52 (13.87) | 153 (40.80) | 4 (1.07) | 375 (87.62) | *Ref.* |
| Yes | 53 (100.00) | 0 (0.00) | 0 (0.00) | 0 (0.00) | 53 (12.38) | -** |
| **Unsafe** |  |  |  |  |  |  |
| No | 133 (40.80) | 36 (11.04) | 153 (46.93) | 4 (1.23) | 326 (76.17) | *Ref.* |
| Yes | 86 (84.31) | 16 (15.69) | 0 (0.00) | 0 (0.00) | 102 (23.83) | 9.33 (5.27, 16.53) |
| **Not effective** |  |  |  |  |  |  |
| No | 197 (48.76) | 50 (12.38) | 153 (37.87) | 4 (0.99) | 404 (94.39) | *Ref.* |
| Yes | 22 (91.67) | 2 (8.33) | 0 (0.00) | 0 (0.00) | 24 (5.61) | 12.28 (2.86, 52.68) |
| **COVID-19 is not dangerous** | | |  |  |  |  |
| No | 217 (51.42) | 48 (11.37) | 153 (36.26) | 4 (0.95) | 422 (98.60) | *Ref.* |
| Yes | 2 (33.33) | 4 (66.67) | 0 (0.00) | 0 (0.00) | 6 (1.40) | 1.18 (0.33, 4.29) |
| **Fear of infection** |  |  |  |  |  |  |
| No | 165 (44.12) | 52 (13.90) | 153 (40.91) | 4 (1.07) | 374 (87.38) | *Ref.* |
| Yes | 54 (100.00) | 0 (0.00) | 0 (0.00) | 0 (0.00) | 54 (12/62) | -** |
| **Against the principle of vaccination in general** | | | | | | |
| No | 164 (45.05) | 43 (11.81) | 153 (42.03) | 4 (1.10) | 364 (85.05) | *Ref.* |
| Yes | 55 (85.94) | 9 (14.06) | 0 (0.00) | 0 (0.00) | 64 (14.95) | 8.49 (4.10, 17.57) |
| **Religious reasons** |  |  |  |  |  |  |
| No | 218 (51.29) | 50 (11.76) | 153 (36.00) | 4 (0.94) | 425 (99.30) | *Ref.* |
| Yes | 1 (33.33) | 2 (66.67) | 0 (0.00) | 0 (0.00) | 3 (0.70) | 1.18 (0.19, 7.23) |
| **Traditional beliefs** |  |  |  |  |  |  |
| No | 211 (50.24) | 52 (12.38) | 153 (36.43) | 4 (0.95) | 420 (98.13) | *Ref.* |
| Yes | 8 (100.00) | 0 (0.00) | 0 (0.00) | 0 (0.00) | 8 (1.87) | -** |
| **I believe in traditional and local medicine** | | | |  |  |  |
| No | 218 (51.05) | 52 (12.18) | 153 (35.83) | 4 (0.94) | 427 (99.77) | *Ref.* |
| Yes | 1 (100.00) | 0 (0.00) | 0 (0.00) | 0 (0.00) | 1 (0.23) | -** |
| **Other reasons** |  |  |  |  |  |  |
| No | 197 (49.00) | 48 (11.94) | 153 (38.06) | 4 (1.00) | 402 (93.93) | *Ref.* |
| Yes | 22 (84.62) | 4 (15.38) | 0 (0.00) | 0 (0.00) | 26 (6.07) | 6.47 (2.21, 18.88) |
| **Without reason** |  |  |  |  |  |  |
| No | 219 (89.75) | 25 (10.25) | 0 (0.00) | 0 (0.00) | 244 (57.01) | *Ref.* |
| Yes | 0 (0.00) | 27 (14.67) | 153 (83.15) | 4 (2.17) | 184 (42.99) | -** |

*, Based on univariate ordinal logistic regression

**, OR could not be calculated due to frequency of categories with zero subjects.

OR: Odds ratio; CI: Confidence interval; Ref.: Reference category

Supplementary Table 7: Multivariate ordered logistic regression to for independent risk factors against COVID-19 vaccination in the internally displaced persons

| **Variable** | **aOR (95% CI)** |
| --- | --- |
| **Age group (year)** |  |
| 12 to 19 | *Ref.* |
| 19 to 45 | 0.04 (0.01, 0.20) |
| 46 to 65 | 0.02 (0.00, 0.10) |
| 65 to 98 | 0.01 (0.00, 0.03) |
| **Gender** |  |
| Male | *Ref.* |
| Female | 2.36 (1.37, 4.07) |
| **Nationality** |  |
| Kurd | *Ref.* |
| Arab | 3.38 (1.56, 7.29) |
| Turkman | 4.02 (1.65, 9.77) |
| **Governate** |  |
| Erbil | *Ref.* |
| Ninawa | 1.92 (0.88, 4.18) |
| **Education level** |  |
| Illiterate | *Ref.* |
| Diploma or less | 0.50 (0.26, 0.96) |
| University | 0.26 (0.10, 0.67) |
| **Occupation** |  |
| Health and medical fields | *Ref.* |
| Non-office worker | 2.71 (1.18, 6.23) |
| Retired | 94.65 (12.96, 690.99) |
| Other | 3.75 (1.75, 8.01) |
| **Factors leading to avoid COVID-19 vaccination** |  |
| Unsafe  No  Yes |  |
|  | *Ref.* |
|  | 9.28 (4.71, 18.27) |
| Not effective  No  Yes |  |
|  | *Ref.* |
|  | 20.07 (3.98, 101.06) |
| Against the principle of vaccination in general  No  Yes |  |
|  | *Ref.* |
|  | 16.19 (6.82, 38.43) |
| Other reasons  No  Yes |  |
|  | *Ref.* |
|  | 14.01 (4.17, 47.05) |

aOR: Adjusted odds ratio; CI: Confidence interval; Ref.: Reference category

Supplementary Table 8: Distribution of baseline characteristics of the refugee subjects according to number of COVID-19 vaccination doses

| **Variables** | **COVID-19 vaccination status** | | | | **Total (%)** |  | **OR* (95% CI)** |
| --- | --- | --- | --- | --- | --- | --- | --- |
|  | **No vaccination** | **One dose** | **Two doses** | **Three doses** |  |  |  |
| **Age group (year)** |  |  |  |  |  |  |  |
| 12 to 18 | 77 (70.64) | 12 (11.01) | 20 (18.35) | 0 (0.00) | 109 (17.67) |  | *Ref.* |
| 19 to 45 | 177 (52.84) | 23 (6.87) | 130 (38.81) | 5 (1.49) | 335 (54.29) |  | 0.42 (0.27, 0.67) |
| 46 to 65 | 62 (42.47) | 14 (9.59) | 66 (45.21) | 4 (2.74) | 146 (23.66) |  | 0.29 (0.17, 0.48) |
| 65 to 98 | 18 (66.67) | 2 (7.41) | 7 (25.93) | 0 (0.00) | 27 (4.38) |  | 0.79 (0.33, 1.91) |
| **Gender** |  |  |  |  |  |  |  |
| Male | 169 (50.75) | 27 (8.11) | 129 (38.74) | 8 (2.40) | 333 (53.97) |  | *Ref.* |
| Female | 165 (58.10) | 24 (8.45) | 94 (33.10) | 1 (0.35) | 284 (46.03) |  | 1.39 (1.02, 1.90) |
| **Governate** |  |  |  |  |  |  |  |
| Erbil | 130 (51.59) | 32 (12.70) | 83 (32.94) | 7 (2.78) | 252 (40.84) |  | *Ref.* |
| Sulaimani | 85 (81.73) | 5 (4.81) | 14 (13.46) | 0 (0.00) | 104 (16.86) |  | 4.02 (2.32, 6.97) |
| Duhok | 63 (48.84) | 5 (3.88) | 60 (46.51) | 1 (0.78) | 129 (20.91) |  | 0.79 (0.52, 1.19) |
| Kirkuk | 8 (34.78) | 4 (17.39) | 11 (47.83) | 0 (0.00) | 23 (3.73) |  | 0.61 (0.28, 1.33) |
| Ninawa | 48 (44.04) | 5 (4.59) | 55 (50.46) | 1 (0.92) | 109 (17.67) |  | 0.66 (0.42, 1.01) |
| **Nationality** |  |  |  |  |  |  |  |
| Kurd | 123 (50.62) | 27 (11.11) | 93 (38.27) | 0 (0.00) | 243 (39.38) |  | *Ref.* |
| Arab | 194 (56.23) | 21 (6.09) | 122 (35.36) | 8 (2.32) | 345 (55.92) |  | 1.11 (0.81, 1.53) |
| Assyrian | 6 (75.00) | 0 (0.00) | 2 (25.00) | 0 (0.00) | 8 (1.30) |  | 2.54 (0.50, 12.81) |
| Turkman | 9 (52.94) | 3 (17.65) | 5 (29.41) | 0 (0.00) | 17 (2.76) |  | 1.21 (0.48, 3.05) |
| Other | 2 (50.00) | 0 (0.00) | 1 (25.00) | 1 (25.00) | 4 (0.65) |  | 0.45 (0.05, 4.36) |
| **Religion** |  |  |  |  |  |  |  |
| Muslim | 285 (55.88) | 47 (9.22) | 169 (33.14) | 9 (1.76) | 510 (82.66) |  | *Ref.* |
| Yazedy | 37 (43.02) | 4 (4.65) | 45 (52.23) | 0 (0.00) | 86 (13.94) |  | 0.57 (0.36, 0.88) |
| Christian | 11 (55.00) | 0 (0.00) | 9 (45.00) | 0 (0.00) | 20 (3.24) |  | 0.84 (0.35, 2.05) |
| Other | 1 (100.00) | 0 (0.00) | 0 (0.00) | 0 (0.00) | 1 (0.16) |  | -** |
| **Marital status** |  |  |  |  |  |  |  |
| Married | 193 (48.61) | 33 (8.31) | 165 (41.56) | 6 (1.51) | 397 (64.34) |  | *Ref.* |
| Single | 127 (67.20) | 17 (8.99) | 42 (22.22) | 3 (1.59) | 189 (30.63) |  | 2.21 (1.55, 3.15) |
| Divorced | 13 (46.43) | 1 (3.57) | 14 (50.00) | 0 (0.00) | 28 (4.54) |  | 0.86 (0.41, 1.80) |
| Other | 1 (33.33) | 0 (0.00) | 2 (66.67) | 0 (0.00) | 3 (0.49) |  | 0.46 (0.05, 4.48) |
| **Education** |  |  |  |  |  |  |  |
| Illiterate | 98 (49.75) | 21 (10.66) | 76 (38.58) | 2 (1.02) | 197 (31.93) |  | *Ref.* |
| Diploma or less | 205 (57.42) | 28 (7.84) | 118 (33.05) | 6 (1.68) | 357 (57.86) |  | 1.28 (0.92, 1.80) |
| University | 31 (49.21) | 2 (3.17) | 29 (46.03) | 1 (1.59) | 63 (10.21) |  | 0.84 (0.49, 1.47) |
| **Occupation** |  |  |  |  |  |  |  |
| Health and medical fields | 1 (8.33) | 1 (8.33) | 10 (83.33) | 0 (0.00) | 12 (1.94) |  | *Ref.* |
| Office worker | 8 (44.44) | 1 (5.56) | 8 (44.44) | 1 (5.56) | 18 (2.92) |  | 3.60 (0.81, 16.01) |
| Non-office worker | 18 (39.13) | 3 (6.52) | 24 (52.17) | 1 (2.17) | 46 (7.46) |  | 3.17 (0.86, 11.68) |
| Military and security | 1 (5.26) | 4 (21.05) | 14 (73.68) | 0 (0.00) | 19 (3.08) |  | 1.30 (0.30, 5.62) |
| Student | 80 (66.67) | 14 (11.67) | 24 (20.00) | 2 (1.67) | 120 (19.45) |  | 10.88 (3.15, 37.58) |
| Retired | 7 (53.85) | 1 (7.69) | 5 (38.46) | 0 (0.00) | 13 (2.11) |  | 6.04 (1.24, 29.45) |
| Others | 219 (56.30) | 27 (6.94) | 138 (35.48) | 5 (1.29) | 389 (63.05) |  | 6.48 (1.96, 21.47) |
| **Health status** |  |  |  |  |  |  |  |
| Positive chronic disease | 59 (50.82) | 9 (7.76) | 45 (38.79) | 3 (2.59) | 116 (18.80) |  | *Ref.* |
| Healthy | 275 (54.89) | 42 (8.38) | 178 (35.53) | 6 (1.20) | 501 (81.20) |  | 1.21 (0.82, 1.79) |

*, Based on univariate ordinal logistic regression

**, OR could not be calculated due to frequency of categories with zero subjects.

OR: Odds ratio; CI: Confidence interval; Ref.: Reference category

Supplementary Table 9: Distribution of possible barriers of COVID-19 vaccination coverage in the refuge subjects according to number of doses

| **Variables** | **COVID-19 vaccination status** | | | | **Total (%)** | **OR* (95% CI)** |
| --- | --- | --- | --- | --- | --- | --- |
|  | **No vaccination** | **One dose** | **Two doses** | **Three doses** |  |  |
| **Side effects** | |  |  |  |  |  |
| No | 256 (47.50) | 51 (9.46) | 223 (41.37) | 9 (1.67) | 539 (87.36) | *Ref.* |
| Yes | 78 (100.00) | 0 (0.0) | 0 (0.00) | 0 (0.00) | 78 (12.64) | -** |
| **Unsafe** |  |  |  |  |  |  |
| No | 218 (44.58) | 39 (7.98) | 223 (45.60) | 9 (1.84) | 489 (79.25) | *Ref.* |
| Yes | 116 (90.63) | 12 (9.38) | 0 (0.00) | 0 (0.00) | 128 (20.75) | 13.39 (7.22, 24.84) |
| **Not effective** |  |  |  |  |  |  |
| No | 262 (48.16) | 50 (9.19) | 223 (40.99) | 9 (1.65) | 544 (88.17) | *Ref.* |
| Yes | 72 (98.63) | 1 (1.37) | 0 (0.00) | 0 (0.00) | 73 (11.83) | 78.44 (10.82, 568.44) |
| **COVID-19 is not dangerous** | | |  |  |  |  |
| No | 302 (51.62) | 51 (8.72) | 223 (38.12) | 9 (1.54) | 585 (94.81) | *Ref.* |
| Yes | 32 (100.00) | 0 (0.00) | 0 (0.00) | 0 (0.00) | 32 (5.19) | -** |
| **Fear of infection** |  |  |  |  |  |  |
| No | 233 (45.24) | 50 (9.71) | 223 (43.30) | 9 (1.75) | 515 (83.47) | *Ref.* |
| Yes | 101 (99.02) | 1 (0.98) | 0 (0.00) | 0 (0.00) | 102 (16.53) | 123.41 (17.10, 891.28) |
| **Against the principle of vaccination in general** | | | | | | |
| No | 309 (53.93) | 32 (5.58) | 223 (38.92) | 9 (1.57) | 573 (92.87) | *Ref.* |
| Yes | 25 (56.82) | 19 (43.18) | 0 (0.00) | 0 (0.00) | 44 (7.13) | 1.88 (1.07, 3.31) |
| **Religious reasons** |  |  |  |  |  |  |
| No | 334 (54.22) | 50 (8.12) | 223 (36.20) | 9 (1.46) | 616 (99.84) | *Ref.* |
| Yes | 0 (0.00) | 1 (100.00) | 0 (0.00) | 0 (0.00) | 1 (0.16) | 0.71 (0.04, 11.58) |
| **Traditional beliefs** |  |  |  |  |  |  |
| No | 325 (53.45) | 51 (8.39) | 223 (36.68) | 9 (1.48) | 608 (98.54) | *Ref.* |
| Yes | 9 (100.00) | 0 (0.00) | 0 (0.00) | 0 (0.00) | 9 (1.46) | -** |
| **I believe in traditional and local medicine** | | | | | | |
| No | 333 (54.15) | 50 (8.13) | 223 (36.26) | 9 (1.46) | 615 (99.68) | *Ref.* |
| Yes | 1 (50.00) | 1 (50.00) | 0 (0.00) | 0 (0.00) | 2 (0.32) | 1.52 (0.14, 16.98) |
| **Other reasons** |  |  |  |  |  |  |
| No | 291 (50.87) | 49 (8.57) | 223 (38.99) | 9 (1.57) | 572 (92.71) | *Ref.* |
| Yes | 43 (95.56) | 2 (4.44) | 0 (0.00) | 0 (0.00) | 45 (7.29) | 21.55 (5.18, 89.68) |
| **Without reason** |  |  |  |  |  |  |
| No | 334 (90.76) | 34 (9.24) | 0 (0.00) | 0 (0.00) | 368 (59.64) | *Ref.* |
| Yes | 0 (0.00) | 17 (6.83) | 223 (89.56) | 9 (3.61) | 249 (40.36) | -** |

*, Based on univariate ordinal logistic regression

**, OR could not be calculated due to frequency of categories with zero subjects.

OR: Odds ratio; CI: Confidence interval; Ref.: Reference category

Supplementary Table 10: Multivariate ordered logistic regression for independent risk factors against COVID-19 vaccination in the refugee subjects

| **Variable** | **aOR (95% CI)** |
| --- | --- |
| **Age group (year)** |  |
| 12 to 19 | *Ref.* |
| 46 to 65 | 0.58 (0.33, 1.02) |
| **Maritial status** |  |
| Married | *Ref.* |
| Single | 3.53 (1.93, 6.46) |
| **Governate** |  |
| Erbil | *Ref.* |
| Sulaimani | 6.67 (3.29, 13.49) |
| **Occupation** |  |
| Health and medical fields | *Ref.* |
| Retired | 5.48 (1.20, 24.92) |
| Other | 1.71 (0.99, 2.93) |
| **Factors leading to avoid COVID-19 vaccination** |  |
| Unsafe  No  Yes |  |
|  | *Ref.* |
|  | 56.02 (26.83, 116.99) |
| Not effective  No  Yes |  |
|  | *Ref.* |
|  | 303.28 (38.60, 2382.88) |
| Fear of infection  No  Yes |  |
|  | *Ref.* |
|  | 921.52 (121.76, 6974.44) |
| Against the principle of vaccination in general  No  Yes |  |
|  | *Ref.* |
|  | 15.32 (7.51, 31.27) |
| Other reasons  No  Yes |  |
|  | *Ref.* |
|  | 77.50 (16.75, 358.46) |

aOR: Adjusted odds ratio; CI: Confidence interval; Ref.: Reference category
