# Supplementary material for: COVID-19 Vaccination Among Diverse Population Groups in the Northern Governorates of Iraq
Source: Int J Public Health. 2023 Nov 28;68:1605736. doi: 10.3389/ijph.2023.1605736 (PMC10713705; doi:10.3389/ijph.2023.1605736)
Supplement: Supplementary file 7 [file Table4.docx]

Supplementary Table 4: Multivariate ordered logistic regression to for independent risk factors against COVID-19 vaccination in the host communities

| **Variable** | **aOR (95% CI)** |
| --- | --- |
| **Age group (year)** |  |
| 12 to 19 | *Ref.* |
| 19 to 45 | 0.22 (0.14, 0.36) |
| 46 to 65 | 0.15 (0.09, 0.26) |
| 65 to 98 | 0.17 (0.09, 0.31) |
| **Religion** |  |
| Muslim | *Ref.* |
| Christian | 0.42 (0.20, 0.90) |
| **Governate** |  |
| Erbil | *Ref.* |
| Sulaimani | 2.68 (2.14, 3.37) |
| Ninawa | 1.86 (1.47, 2.36) |
| **Education level** |  |
| Illiterate | *Ref.* |
| Diploma or less | 0.53 (0.41, 0.68) |
| University | 0.32 (0.23, 0.45) |
| **Occupation** |  |
| Health and medical fields | *Ref.* |
| Office worker | 1.43 (0.97, 2.13) |
| Non-office worker | 2.13 (1.51, 3.02) |
| Student | 2.95 (1.97, 4.43) |
| Other | 3.32 (2.50, 4.41) |
| **Factors leading to avoid COVID-19 vaccination** |  |
| Unsafe  No  Yes |  |
|  | *Ref.* |
|  | 33.10 (24.48, 44.75) |
| Not effective  No  Yes |  |
|  | *Ref.* |
|  | 18.11 (11.41, 28.77) |
| Corona disease is not dangerous  No  Yes |  |
|  | *Ref.* |
|  | 42.42 (24.03, 74.87) |
| Fear of infection  No  Yes |  |
|  | *Ref.* |
|  | 339.24 (155.83, 738.54) |
| Against the principle of vaccination in general  No  Yes |  |
|  | *Ref.* |
|  | 28.80 (20.45, 40.56) |
| Religious reasons  No  Yes |  |
|  | *Ref.* |
|  | 11.14 (3.26, 38.10) |
| Other reasons  No  Yes |  |
|  | *Ref.* |
|  | 103.78 (60.07, 179.27) |

aOR: Adjusted odds ratio; CI: Confidence interval
