# Supplementary material for: COVID-19 Vaccination Among Diverse Population Groups in the Northern Governorates of Iraq
Source: Int J Public Health. 2023 Nov 28;68:1605736. doi: 10.3389/ijph.2023.1605736 (PMC10713705; doi:10.3389/ijph.2023.1605736)
Supplement: Supplementary file 8 [file Table6.docx]

Supplementary Table 6: Distribution of possible barriers of COVID-19 vaccination coverage in the internally displaced persons according to number of doses

| **Variables** | **COVID-19 vaccination status** | | | | **Total (%)** | **OR* (95% CI)** |
| --- | --- | --- | --- | --- | --- | --- |
|  | **No vaccination** | **One dose** | **Two doses** | **Three doses** |  |  |
| **Side effects** | |  |  |  |  |  |
| No | 166 (44.27) | 52 (13.87) | 153 (40.80) | 4 (1.07) | 375 (87.62) | *Ref.* |
| Yes | 53 (100.00) | 0 (0.00) | 0 (0.00) | 0 (0.00) | 53 (12.38) | -** |
| **Unsafe** |  |  |  |  |  |  |
| No | 133 (40.80) | 36 (11.04) | 153 (46.93) | 4 (1.23) | 326 (76.17) | *Ref.* |
| Yes | 86 (84.31) | 16 (15.69) | 0 (0.00) | 0 (0.00) | 102 (23.83) | 9.33 (5.27, 16.53) |
| **Not effective** |  |  |  |  |  |  |
| No | 197 (48.76) | 50 (12.38) | 153 (37.87) | 4 (0.99) | 404 (94.39) | *Ref.* |
| Yes | 22 (91.67) | 2 (8.33) | 0 (0.00) | 0 (0.00) | 24 (5.61) | 12.28 (2.86, 52.68) |
| **COVID-19 is not dangerous** | | |  |  |  |  |
| No | 217 (51.42) | 48 (11.37) | 153 (36.26) | 4 (0.95) | 422 (98.60) | *Ref.* |
| Yes | 2 (33.33) | 4 (66.67) | 0 (0.00) | 0 (0.00) | 6 (1.40) | 1.18 (0.33, 4.29) |
| **Fear of infection** |  |  |  |  |  |  |
| No | 165 (44.12) | 52 (13.90) | 153 (40.91) | 4 (1.07) | 374 (87.38) | *Ref.* |
| Yes | 54 (100.00) | 0 (0.00) | 0 (0.00) | 0 (0.00) | 54 (12/62) | -** |
| **Against the principle of vaccination in general** | | | | | | |
| No | 164 (45.05) | 43 (11.81) | 153 (42.03) | 4 (1.10) | 364 (85.05) | *Ref.* |
| Yes | 55 (85.94) | 9 (14.06) | 0 (0.00) | 0 (0.00) | 64 (14.95) | 8.49 (4.10, 17.57) |
| **Religious reasons** |  |  |  |  |  |  |
| No | 218 (51.29) | 50 (11.76) | 153 (36.00) | 4 (0.94) | 425 (99.30) | *Ref.* |
| Yes | 1 (33.33) | 2 (66.67) | 0 (0.00) | 0 (0.00) | 3 (0.70) | 1.18 (0.19, 7.23) |
| **Traditional beliefs** |  |  |  |  |  |  |
| No | 211 (50.24) | 52 (12.38) | 153 (36.43) | 4 (0.95) | 420 (98.13) | *Ref.* |
| Yes | 8 (100.00) | 0 (0.00) | 0 (0.00) | 0 (0.00) | 8 (1.87) | -** |
| **I believe in traditional and local medicine** | | | |  |  |  |
| No | 218 (51.05) | 52 (12.18) | 153 (35.83) | 4 (0.94) | 427 (99.77) | *Ref.* |
| Yes | 1 (100.00) | 0 (0.00) | 0 (0.00) | 0 (0.00) | 1 (0.23) | -** |
| **Other reasons** |  |  |  |  |  |  |
| No | 197 (49.00) | 48 (11.94) | 153 (38.06) | 4 (1.00) | 402 (93.93) | *Ref.* |
| Yes | 22 (84.62) | 4 (15.38) | 0 (0.00) | 0 (0.00) | 26 (6.07) | 6.47 (2.21, 18.88) |
| **Without reason** |  |  |  |  |  |  |
| No | 219 (89.75) | 25 (10.25) | 0 (0.00) | 0 (0.00) | 244 (57.01) | *Ref.* |
| Yes | 0 (0.00) | 27 (14.67) | 153 (83.15) | 4 (2.17) | 184 (42.99) | -** |

*, Based on univariate ordinal logistic regression

**, OR could not be calculated due to frequency of categories with zero subjects.

OR: Odds ratio; CI: Confidence interval; Ref.: Reference category
