# Supplementary material for: COVID-19 Vaccination Among Diverse Population Groups in the Northern Governorates of Iraq
Source: Int J Public Health. 2023 Nov 28;68:1605736. doi: 10.3389/ijph.2023.1605736 (PMC10713705; doi:10.3389/ijph.2023.1605736)
Supplement: Supplementary file 9 [file DataSheet1.docx]

**Supplementary Material 1.**

***Study sample and sampling method:***

The study sample comprised different sub-populations (the general population, IDPs, and refugees) in each governorate. To select the sample, every household in the selected communities was identified on a sketch map and the household list of each area under study. From these lists a small number of households were randomly selected to participate using a household form. The sample size was 550 households, which was calculated following the recommendations by WHO as below:

A: Number of strata = 5 (one survey in each governorate)

B: Effective sample size (primary number) = 385

The primary sample size was calculated using N = Z^2^_α_×P×(1 − P) /d^2^, where P=0.50, α = 0.05 and Z_α_ = 1.96, and the estimated acceptable margin of error for proportion “d” was set as 0.05.

C: Required data were collected from a household selected as the starting point from the map or the list of the households and their neighbors (named cluster) to get at least m=7 respondents per cluster, assuming an intra cluster correlation coefficient of about 0.17 (0.167), so the design effect was 2 [23].

Total target respondents with completed questionnaires:

Number of target sample = A × B × C = (5 × 385 × 2) = 3850

Then, the total clusters in the survey = (A × B × C) /𝑚 = (5 × 385 × 2) /7 = 550

Supplementary Table 1 indicated the number of required participants and clusters according to governorates and the type of subpopulations.

Supplementary Table 1. The number of required sample size and the number of clusters according to governorates

| **Governorate** | **Strata** | **Number of people** | **sample size** | **Number of clusters** |
| --- | --- | --- | --- | --- |
| Dohuk | General Population | 616600 | 260 | 37 |
|  | IDPs in camp | 140640 | 59 | 8 |
|  | IDPs outside camp | 108525 | 44 | 7 |
|  | Refugees in camp | 55687 | 24 | 3 |
|  | Refugees outside camp | 30469 | 14 | 2 |
|  | **Total** | **951921** | **401** | **57** |
| Sulaymaniyah | General Population | 2159800 | 909 | 130 |
|  | IDPs in camp | 11550 | 14 | 2 |
|  | IDPs outside camp | 128268 | 54 | 8 |
|  | Refugees in camp | 9641 | 14 | 2 |
|  | Refugees outside camp | 22698 | 14 | 2 |
|  | **Total** | **2333277** | **982** | **140** |
| Erbil | General Population | 1845200 | 777 | 111 |
|  | IDPs in camp | 13295 | 14 | 2 |
|  | IDPs outside camp | 219240 | 92 | 13 |
|  | Refugees in camp | 22098 | 14 | 2 |
|  | Refugees outside camp | 98841 | 42 | 6 |
|  | **Total** | **2198674** | **962** | **132** |
| Kirkuk | General Population | 839100 | 354 | 51 |
|  | IDPs in camp | 0 | 0 | 0 |
|  | IDPs outside camp | 91080 | 38 | 6 |
|  | Refugees in camp | 0 | 0 | 0 |
|  | Refugees outside camp | 771 | 0 | 0 |
|  | **Total** | **930951** | **392** | **56** |
| Ninawa | General Population | 2473700 | 1041 | 149 |
|  | IDPs in camp | 45225 | 19 | 3 |
|  | IDPs outside camp | 208278 | 87 | 13 |
|  | Refugees in camp | 0 | 0 | 0 |
|  | Refugees outside camp | 802 | 0 | 0 |
|  | **Total** | **2728005** | **1149** | **165** |
| **Total** | | **9142828** | **3850** | **550** |

However, to increase the power of the study, a total of 4564 respondents from all the governorates were recruited for the purpose of the present study. The survey had the power to detect a minimum coverage rate of 8% with considering a maximum marginal error of 1/10 proportion (d= 1/10 proportion).

***Data collection procedure:***

In this study, over 550 clusters were selected and stratified by governorates. After randomly choosing the initial HHs, sampling from eligible respondents from neighboring households was continued until at least 7 respondents were surveyed per cluster. The number of the completed questionnaires varied per cluster, but the minimum average was 7.

Data were collected using Kobo Toolbox. Choosing the cluster was performed through a number of steps. First, the cumulative population was obtained from populations of health center catchment areas. Second, the population was divided (in general and then the total number of IDPs who lived inside camps per governorate, each by the number of allocated clusters to obtain K) for example in general population of Sulaimani, K=2,336,191/130 = 17,971 for general population. The location of the first cluster was found by finding a random number between 1 and K (1- 17971). In the cumulative list, the first cluster was located in the area of 13081, which was Sarchnar district here (in Sulaimani). For the second cluster, we summated the 13081 + 17971 = 31052. So, in cumulative list, we found 31052 for the second cluster, and 49024, 66966, 84968, …, and 2331468 were found for the rest of the 130 clusters. The 130^th^ cluster was located in Salhan in the sampling cumulative list. The same process was followed for the IDP and refugee populations living inside the camps. In each governorate one to three distributed teams (one team leader and two to six data collectors) gathered the required data.
