# Supplementary material for: COVID-19 Vaccination Among Diverse Population Groups in the Northern Governorates of Iraq
Source: Int J Public Health. 2023 Nov 28;68:1605736. doi: 10.3389/ijph.2023.1605736 (PMC10713705; doi:10.3389/ijph.2023.1605736)
Supplement: Supplementary file 10 [file Table10.docx]

Supplementary Table 10: Multivariate ordered logistic regression for independent risk factors against COVID-19 vaccination in the refugee subjects

| **Variable** | **aOR (95% CI)** |
| --- | --- |
| **Age group (year)** |  |
| 12 to 19 | *Ref.* |
| 46 to 65 | 0.58 (0.33, 1.02) |
| **Maritial status** |  |
| Married | *Ref.* |
| Single | 3.53 (1.93, 6.46) |
| **Governate** |  |
| Erbil | *Ref.* |
| Sulaimani | 6.67 (3.29, 13.49) |
| **Occupation** |  |
| Health and medical fields | *Ref.* |
| Retired | 5.48 (1.20, 24.92) |
| Other | 1.71 (0.99, 2.93) |
| **Factors leading to avoid COVID-19 vaccination** |  |
| Unsafe  No  Yes |  |
|  | *Ref.* |
|  | 56.02 (26.83, 116.99) |
| Not effective  No  Yes |  |
|  | *Ref.* |
|  | 303.28 (38.60, 2382.88) |
| Fear of infection  No  Yes |  |
|  | *Ref.* |
|  | 921.52 (121.76, 6974.44) |
| Against the principle of vaccination in general  No  Yes |  |
|  | *Ref.* |
|  | 15.32 (7.51, 31.27) |
| Other reasons  No  Yes |  |
|  | *Ref.* |
|  | 77.50 (16.75, 358.46) |

aOR: Adjusted odds ratio; CI: Confidence interval; Ref.: Reference category
